# Diverse nature of ClpX degradation motifs in *Streptococcus mutans*

**DOI:** 10.1128/spectrum.03457-23

**Published:** 2023-12-05

**Authors:** Vivek Gurung, Saswati Biswas, Indranil Biswas

**Affiliations:** 1 Department of Microbiology, Molecular Genetics, and Immunology, University of Kansas Medical Center, Kansas City, Kansas, USA; The Ohio State University College of Dentistry, Columbus, Ohio, USA

**Keywords:** AAA+ ATPase, clp proteins, ClpX substrates, degradation motifs, HSP100, proteolysis

## Abstract

Protein homeostasis is an essential process that depends on concerted effort of different proteins involved in proper protein folding, deciding the fate of misfolded proteins by either refolding them into their proper conformation or degrading the misfolded proteins. In Gram-positive bacteria, AAA+ ATPases such as ClpX, ClpC, and ClpE interact with the proteolytic ClpP and degrade misfolded proteins. Clp ATPases also maintain amounts of different proteins based on the cellular need. In streptococci, these Clp ATPases play a crucial role in various physiological processes that contribute to virulence, cell growth and division, stress tolerance, competence development, and biofilm formation. Among the Clp ATPase complexes present in low G + C Gram-positive bacteria, ClpX/P is the major proteolytic system. In contrast to numerous ClpX/P substrates identified in *Escherichia coli* and other bacteria, very little is known about the identity of the ClpX/P substrates in streptococci. Using a proteomic approach under late logarithmic growth condition, we screened for putative substrates that might be degraded by ClpX/P; several potential substrates were identified and verified by other methods. SpxA2 is one such candidate protein, and by Western blot, we confirmed that it indeed recognizes SpxA2. We further identified Ala-Ala-Leu, located at the C-terminal of SpxA2, as a ClpX/P degradation signal. Using a green fluorescent protein reporter system, we further confirmed several ClpX/P degradation signals. Furthermore, we found that the zinc-binding domain of ClpX is needed for substrate recognition. Our *in vitro* data indicate that the adaptor or other accessory factors might be needed for the ClpX/P-mediated substrate recognition or degradation. Additional investigations are needed to determine the identity of these factors in the cell.

**IMPORTANCE:**

Cytoplasmic Clp-related proteases play a major role in maintaining cellular proteome in bacteria. ClpX/P is one such proteolytic complex that is important for conserving protein homeostasis. In this study, we investigated the role of ClpX/P in *Streptococcus mutans*, an important oral pathogen. We identified several putative substrates whose cellular levels are regulated by ClpX/P in *S. mutans* and subsequently discovered several recognition motifs that are critical for degradation. Our study is the first comprehensive analysis of determining ClpX/P motifs in streptococci. We believe that identifying the substrates that are regulated by ClpX/P will enhance our understanding about virulence regulation in this important group of pathogens.

## INTRODUCTION

The proteome composition of a cell defines its proper cellular function and its fate. Proteome composition maintenance involves controlled transcription and translation, proper protein folding, refolding of misfolded proteins, disaggregation of aggregated proteins, and proteolysis of both correctly folded and misfolded proteins. In bacteria, a complex network of molecular chaperones (GroEL, DnaK, trigger factor, ClpB), co-chaperones (GroES, DnaJ), and proteases (FtsH, AAA+ proteases) form the protein quality control network dedicated to proteome composition maintenance at the translational and post-translational levels (1). Various AAA+ (ATPases associated with cellular activities) proteases, including ClpA/P, ClpC/P, ClpE/P, ClpX/P, HslUV, FtsH, and Lon proteases, are involved in the bacterial proteolysis (2). Some of these proteases fall into two groups of the Clp/HSP100 family. While the class I proteins (ClpA/P, ClpC/P, and ClpE/P) contain two ATPase domains, the class II proteins, such as ClpX and HslU, encode a single AAA+ ATPase domain (3). FtsH and Lon proteases do not fall into the HSP100 family. Using ATP hydrolysis as a driving force, these proteases degrade various proteins in the cell and thus modulate the proteome composition throughout the growth (4).

Since proteolysis is an irreversible and energy-consuming process, this process is strictly regulated to prevent undesired protein degradation. Clp proteolytic complexes are generally large and consist of an unfoldase component (ClpA, ClpC, ClpE, and ClpX) and a proteolytic component (ClpP) (5 – 7). Structurally, these Clp ATPases form a hexameric homocomplex that interacts with the tetradecameric ClpP homocomplex to create an active hetero-complex (ClpA/P, ClpC/P, ClpE/P, and ClpX/P) (5). In contrast, Lon and FtsH proteases encode both the substrate recognizing and the proteolytic domains (8, 9).

Clp ATPases play a crucial role in specificity for substrate selection and degradation. Not all cellular proteome is subjected to Clp-mediated degradation. Clp ATPases identify the substrates, unfold them using energy from the ATP hydrolysis, and deliver the denatured polypeptides to the ClpP proteolytic chamber for degradation (5, 10). The presence of different Clp ATPases varies between Gram-positive and Gram-negative bacteria. For example, ClpA is strictly present in Gram-negative bacteria such as *Escherichia coli* but absent in Gram-positive bacteria (6). On the other hand, ClpE is generally found in most Gram-positive bacteria, including *Bacillus subtilis*, *Listeria monocytogenes*, *Staphylococcus aureus*, *Streptococcus mutans*, and others but is conspicuously absent in all the Gram-negative bacteria (6, 11).

Among the Clp ATPases, ClpX is significantly smaller in size than the other Clp ATPases, since it encodes only one AAA+ ATPase domain compared to two AAA+ ATPases domains containing Clp ATPases (ClpA, ClpC, and ClpE) (6). Furthermore, unlike other Clp ATPases, whose expressions are induced under various stress conditions, the expression of ClpX during cell growth is largely constant throughout the normal growth conditions as well as under stresses (12, 13). Functionally, one or two hexameric complexes of ClpX ATPases interact with the heptameric ClpP proteolytic complex resulting in either single-capped or double-capped ClpX/P proteolytic complex (14). However, like other Clp ATPases, ClpX also recognizes the substrates, followed by ATP-dependent unfolding and translocation of the substrate to the ClpP proteolytic chamber through the axial pores. ClpX/P can recognize its substrates either directly by binding to the substrate or with the help of additional proteins called adaptor proteins (15, 16). The involvement of these adaptor proteins results in increased substrate diversity. Studies with established systems have shown that most adaptor proteins interact with the N-terminal zinc-binding domain (ZBD) of ClpX (17 – 20). ClpX detects the substrates for direct recognition by identifying distinct short peptide sequences in its substrates that serve as a degradation signal (degradation motif or degron) (10). These degradation motifs can be located at the N-terminal, C-terminal, or a few amino acids away from the N-terminal and C-terminal end (21, 22).

Extensive studies on Gram-negative bacteria such as *E. coli* and *Caulobacter crescentus* have identified numerous ClpX/P substrates (21, 23). In contrast, only a few ClpX/P substrates have also been identified in *B. subtilis*, which is considered a paradigm for Gram-positive bacteria species, and *Staphylococcus aureus* (24 – 29). The identity of ClpX/P substrates is very limited for other Gram-positive bacterial species, including listeria and streptococci. *S. mutans*, an obligate pathogen with a high amount of environmental stress tolerating ability, has gained interest in serving as a model system for Gram-positive bacteria (30). *S. mutans* is the primary causative agent of dental caries. Like other Gram-positive bacteria, ClpX/P is also the major proteolytic system in this organism. Absence of a functional ClpX protein results in growth defects, loss of competence development, reduced virulence, and sensitivity to various stresses in *S. mutans* (31). Since ClpX/P plays a significant role in the physiology of *S. mutans*, identifying its substrates will help us to understand the underlying mechanistic reasoning behind these effects. However, little is known about the identity of the ClpX/P substrates and degradation signals in *S. mutans* (21). We have previously reported that short tripeptide motifs such as Leu, Pro, Phe (LPF) and Glu, Leu, Gln (ELQ) located at the ultimate C-terminal or near the C-terminal are recognized by ClpX/P (22, 32).

In this study, we applied a tandem-mass-tag (TMT) labeling-based proteomic approach to screen for proteins accumulated in the ClpX inactivated cells. We hypothesized that the accumulation of some of these proteins results directly due to failure of ClpX/P-mediated degradation. We identified several such putative substrates and confirmed several degradation motifs by Western blot and reporter fusion analyses. We further showed that the recognition of the degradation motifs is dependent on the ZBD of ClpX. Collectively, our study revealed several new degradation motifs in *S. mutans*.

## MATERIALS AND METHODS

### Bacterial strains, plasmids, and growth conditions

Bacterial strains and plasmids used in this study are listed in Table S1. *S. mutans* strains were grown in Todd Hewitt broth supplemented with 0.2% yeast extract (THY) at 37°C under microaerophilic conditions in a candle jar. Antibiotics such as erythromycin (Em; 10  µg/mL) or chloramphenicol (Cm; 5  µg/mL) were added to the THY medium whenever required. *E. coli* strains were grown in Luria-Bertani (LB) broth supplemented with ampicillin (100  µg/mL), Em (500  µg/mL), or Cm (20  µg/mL) whenever required in a shaking incubator at 37°C. Transformations in *S. mutans* and *E. coli* were performed using previously described protocols using the natural competence and heat shock methods, respectively (33, 34). For growth kinetics, overnight culture was sub-cultured (1:20) in THY and grown at 37°C in a 96-well plate covered with a lid. OD_600_ was measured at 30-min intervals for 12 h using a microplate reader (BioTek Synergy).

### Plasmid construction and transformation

To construct vectors expressing *spxA2*, we PCR amplified the respective open reading frame encoding regions from UA159 genomic DNA (for all the primers, see Table S2) and ligated to BamHI- and EcoRI-digested shuttle vector pIBY35 (22). For plasmids expressing green fluorescent protein (GFP) or GFP^sf^ fusion proteins, we PCR amplified the *gfp* or *sf-gfp* gene with different C-terminal motif sequences from pIBW28 (pIBY35::*gfp*) or pIB1F10 (pIBY35::*sf-gfp*) plasmids, respectively (Table S3). The fragments were digested with *Bam*HI and *Pst*I at 37°C and ligated to *Bam*HI- and *Pst*I-digested pIB190 or pIBY35 at 22°C. The ligated product was then transformed into *E. coli* DH5α and selected on erythromycin-containing LB plates. The resultant constructs were verified by PCR and sequencing using vector-specific primers (pIB190F and pIB190R; Table S2). For *clpX* complementation studies, a full-length *clpX* gene with a native ribosome site was amplified from UA159 genomic DNA, digested with *Bam*HI and *Xho*I enzymes, and cloned into *Bam*HI- and *Xho*I-digested pIB166 to generate pIB1F13. To generate ZBD-deleted *clpX*, using a reverse PCR method, the whole pIB1F13 plasmid was amplified such that the part of the *clpX* gene sequence encoding for the N-terminal amino acids from the 10th position to the 60th of ClpX was not amplified (ΔN_10-60_ *clpX*). The PCR product was then treated with polynucleotide kinase at 37°C, followed by ligation at 22°C. The constructs were verified by PCR and sequenced using vector-specific sequencing primers (pIB166F and pIB166R; Table S2).

### Proteomic analysis

TMT-based labeling and proteomic analysis were performed using the same protocol described previously for the wild-type *S. mutans* UA159 strain and its isogenic *ΔclpX* mutant (35). Briefly, overnight grown cultures were sub-cultured (1:20 dilution) in THY medium at 37°C and were harvested when they reached OD_600_ of 1. Cell pellets were washed in phosphate buffered saline (PBS), resuspended in B-PER solution (Thermo Scientific), and lysed using a bead beater. The lysate was centrifuged to remove the cell debris and stored at −20°C until further use. Total protein extracts were reduced, alkylated, and subjected to proteolytic digestion using filter-aided sample preparation, as previously described (36). About 100-µg protein was processed for each sample. After overnight digestion, the peptidomes were harvested by centrifugation, dried down, and resuspended in pure water.

For TMT quantitation, peptidomes were labeled with a TMT reagent (Thermo Scientific) and then purified by solid-phase extraction using Isolute C18(EC) spin columns (BioTage). An equal quantity of each sample was mixed for multidimensional protein identification technology (MudPIT), and offline fractionation (first dimension) was carried out on an XBridge peptide BEH130 C18 column (100 mm by 400 µm; Waters) under basic conditions.

Peptides were fractionated in a single, two-step reversed-phase gradient of buffer A (10 mM ammonium formate, pH ∼10) and buffer B (acetonitrile with 5% buffer A) as follows: 1% to 30% buffer B from 0 to 100 min, 30% to 40% buffer B from 100 to 120 min, at a constant flow rate of 8 µL/min. Twenty-four 5-min fractions were pooled into eight samples (every third fraction) and dried down for liquid chromatography–mass spectrometry (LC-MS). The eight MudPIT fractions were further resolved by acidic reversed-phase LC-MS with data-dependent acquisition (QExactive plus MS system), acquiring MS survey scans at 70,000 resolution and 18 dependent scans per cycle at 17,500 resolutions. Data files from MudPIT were merged and searched with Mascot version 2.6 (Matrix Science) against the *S. mutans* UA159 protein database (4,840 sequences) with a reversed-sequence decoy database search, applying a significance threshold of *P* < 0.05. For protein identification, the peptide mass tolerance was 8 ppm, and the tandem MS (MS/MS) peak tolerance was 0.02 Da, allowing one missed cleavage for identification. TMT ratios were normalized by using the average ratio of all peptides.

### Protein extraction from bacterial culture

Protein extraction from *S. mutans* cultures was carried out as previously described (22). Briefly, the culture was grown at 37°C to the late stationary phase (OD_600_ ∼1.0 to 1.2). The culture was centrifuged at 8,000 rpm for 10 min at 4°C and resuspended in 700 µL of buffer A [25 mM HEPES-NaOH (pH 7.5), 300 mM NaCl, 5% glycerol). The resuspended cells were then lysed using a bead beater (MP Biomedicals) at 5 m/s for 30 s (five times). *E. coli* cultures were similarly grown, collected at their stationary phase, and lysed by sonication (intensity 5 for 2 min with 10 s on and 20 s off). The lysed cell culture was centrifugated, and the clarified supernatant was collected.

### Western blot analysis

After determining the total protein concentration by the Bradford protein estimation method, the whole cell lysates were separated by 13% SDS-PAGE as previously described (22). The separated samples were transferred from the gel to a polyvinylidene difluoride membrane by the wet transfer method at 300 mA for 2 h. The blots were blocked with blocking buffer (5% bovine serum albumin [BSA] in Tris-buffered saline [TBS]) at room temperature for 1 h. The blotted membranes were probed with mouse anti-His antibody (Sigma), rabbit anti-GFP antibody (Sigma), or rabbit anti-FLAG antibody (Sigma) primary antibodies to detect 6× His-tagged proteins (pIB190), GFP/GFP^sf^ fusion proteins, and EmR-FLAG-tagged proteins, respectively. *S. mutans* anti-enolase rabbit polyclonal antibody (Genscript) (32) was used to measure the enolase level as a loading control in some of the experiments before using Em^R^-FLAG as a loading control. Goat anti-mouse-horseradish peroxidase (HRP) (Sigma) or goat anti-rabbit-HRP (Thermo Scientific) was used as secondary antibodies. The same blot was used to measure the protein levels of enolase and the proteins of interest simultaneously by probing the blot with a mixture of primary antibodies (1:1,000 dilution for anti-His, 1:4,000 dilution for anti-GFP, and 1:8,000 for anti-enolase). Since Em^R^-FLAG and GFP fusion proteins have similar molecular weights, the same amount of cell lysate was loaded onto two separate gels and probed with the antibodies. The blots were further developed with Pierce ECL plus reagent (Thermo Scientific), and the signals were detected on X-ray films or the iBright imaging system (Thermo Scientific). Each experiment was repeated at least two times with independently grown cultures. For the kinetic experiments, 20 µg/mL chloramphenicol was added to the culture during its exponential growth phase (OD_600_ ∼0.6). The cells were harvested at different time points, cell lysates were prepared, and Western blot analysis was performed as described above.

### *In vivo* fluorescence assay

*S. mutans* UA159 and its isogenic *clpX* mutant strains expressing GFP^sf^ fusions were grown overnight and sub-cultured to O.D_600_ of 1.2. The cells were harvested, washed in PBS, and diluted to O.D_600_ of 0.2 in PBS. Three hundred microliters of each sample were loaded into a black flat-bottomed 96-well plate. GFP^sf^ fluorescence signals (λ_exi_/λ_emi_ = 467 nm/511  nm) were detected with a BioTek Synergy/H1 microplate reader.

### Protein purification from *E. coli*

To purify GFP^sf^ fusion proteins with the putative degradation signals (AVAA, AAL, ETV, VTL), the constructs were PCR amplified from pIB1F10 clones. The amplified products were digested with *Bam*HI and *Pst*I and ligated to the pASK-IBA43+ expression vector. The constructs were transformed in the *E. coli* DH5α strain, and protein expression was induced by adding 200 µg/L anhydrotetracycline to the shaking culture at OD_600_ of 0.5. After 6-h incubation, the cells were harvested and lysed in buffer B [25 mM HEPES-NaOH (pH 7.5), 250 mM NaCl, 5% glycerol] by sonication. Recombinant proteins were purified using nickel-nitrilotriacetic acid affinity chromatography and dialyzed against buffer C [50 mM HEPES-KOH (pH 7.5), 300 mM KCl, 10% glycerol] and concentrated using a spin concentrator. For the purification of *S. mutans* ClpX protein, the respective gene was cloned in the pET-Duet vector using *Nco*I and *Pst*I, and the construct was introduced into the *E. coli* BL21 strain. Protein expression was induced by adding 1 mM isopropyl β-D-1-thiogalactopyranoside to the shaking culture at OD_600_ of 0.9, followed by incubation for 6 h at 37°C. After harvesting the culture, the cell pellets were lysed in buffer B [25 mM HEPES-KOH (pH 7.5), 250  mM NaCl, 5% glycerol] by sonication. ClpX was purified using the HiTrap MonoQ column (GE Healthcare) and dialyzed against buffer C [50 mM HEPES/KOH (pH 7.5), 300  mM KCl, 10% glycerol]. *S. mutans* ClpP, GFP, and GFP-AVAA were purified as previously described (32). Protein purity was assessed by separation in 12% SDS-PAGE followed by Coomassie blue staining.

### *In vitro* degradation assay

The *in vitro* degradation assays were performed as previously described (22). Briefly, 0.1 µM substrates (GFP^sf^ variants) were added to a 100-µL digestion system consisting of 1 µM ClpX, 1.2 µM ClpP, and an ATP regeneration system (4 mM ATP, 16 mM creatine phosphate, 0.32 mg/mL creatine kinase) pre-incubated at 30°C for 10 min in a digestion buffer [50 mM HEPES/NaOH (pH 7.6), 5 M MgCl_2_, 300 mM KCl, 10% glycerol]. The reactions were carried out in a black flat-bottomed 96-well plate at 30°C for 60 min. GFP fluorescence signals (λ_exi_/λ_emi_ = 467 nm/511 nm) were detected with a BioTek Synergy/H1 microplate reader.

## RESULTS

### *S. mutans* lacking a functional ClpX ATPase leads to alteration of cellular proteins with diverse function

ClpX/P complex plays a crucial role in protein homeostasis and is involved in the regulation of numerous proteins in the cell (21, 37, 38). To gain insight into how ClpX/P maintains protein homeostasis in *S. mutans* during normal late logarithmic growth conditions, we used a proteomic approach to preliminary identify the proteins that are affected by the loss of functional ClpX. We compared the cellular proteome of the wild-type UA159 strain and its isogenic *clpX* deleted (*ΔclpX;* IBSJ4) strain by TMT-labeled substrates as detailed in Materials and Methods. The cell lysates were prepared from late logarithmically grown cultures in the nutrient-rich THY media at 37°C. *S. mutans* strains generally encode approximately 2,000 different proteins, and we have previously found that a typical mass spectrometry analysis of *S. mutans* UA159 strain using TMT labeling and LC/MS separation can successfully identify approximately 1,200 proteins (35). When we compared the proteome of the two strains, we found that 11 proteins accumulated in the mutant when we set the cut-off value of 2.0-fold (Table 1; Tables S1 and S4). When we set the cut-off values to 1.5-fold, we found additional 42 proteins were differentially accumulated in the *ΔclpX* strain. However, when we lowered the cut-off value to 1.2-fold, additional 118 proteins were found (see Table S1). This cut-off value was chosen because we previously reported that SsbA protein, an authentic ClpX substrate, accumulated approximately 1.2-fold times more in the *ΔclpX* strain than the wild-type strain in our TMT proteomic data (32). Thus, we found that nearly 8.5% of the total proteome (171 total) or ~15% among the TMT-identified proteins was increased in the *ΔclpX* mutant. Based on the protein sequence homology and Kyoto Encyclopedia of Genes and Genomes (KEGG) pathway analysis, we found that most of the differentially accumulated proteins were related to carbohydrate metabolism (~16% among the total accumulated), followed by proteins involved in nucleotide (~9%) and amino acid (~7%) metabolisms based on their predicted role. The other categories were membrane transport (6%), transcription (3%), translation (3%), and protein folding (3%). Several cell wall-associated and stress-related proteins also accumulated (~7%) (Table 1; Table S3). Additionally, the rest of the proteins (~29%) belonged to either an unknown category or metabolisms of secondary metabolites. We also observed that the abundance of several proteins was reduced in the *ΔclpX* strain compared to the wild-type strain. When we set the cut-off value to 0.8-fold, we found that the ~133 proteins were differentially reduced in the mutant (Table S1). These proteins are most likely not ClpX substrates. Among these 133 proteins, the abundance of only two proteins was reduced approximately 2.0-fold (Table S1). The first is ComYC encoded by SMU.1984, and the second is a putative glutathione reductase encoded by SMU.140. Accumulation of two other conserved hypothetical proteins (SMU.139 and SMU.1699) was also reduced to nearly 2.0-fold (Table S3).

**TABLE 1:** Top 10 proteins that are most accumulated in Δ*clpX* strain

Protein ID	Description	Locus tag	*ΔclpX/* WT^*a*^	C-terminal a.a.
CDK10767.1	tmRNA		7.03	KNTNSYAVAA
AAN59679.1	Transcriptional regulator SpxA	SMU.2084	5.62	NTAARLRAAL
AAN58666.1	Putative dehydrogenase	SMU.962	4.94	VQLRHILETV
AAN58665.1	Conserved hypothetical protein	SMU.961	4.64	INPELQQYVK
AAN58637.1	Hypothetical protein	SMU.932	3.8	IAWVKEAVTL
AAN57897.1	Tagatose 1,6-aldolase (lacD2)	SMU.116	2.51	SPWTEKVSVG
AAN58638.1	Putative amino acid ABC transporter	SMU.933	2.28	GEDVFNYVTK
AAN58073.1	Sorbitol operon activator	SMU.310	2.11	KLKKRHETKI
AAN58928.1	Conserved hypothetical protein	SMU.1245	2.1	QLLALLVRKS
AAN59670.1	Ribonucleoside-triphosphate reductase	SMU.2074	2.05	STIKNPGHKA

^*a*^ Ratios indicated are the simple ratio.

### Transcription factor SpxA2 encoded by SMU.2084 is a ClpX/P substrate

**Fig 1:**
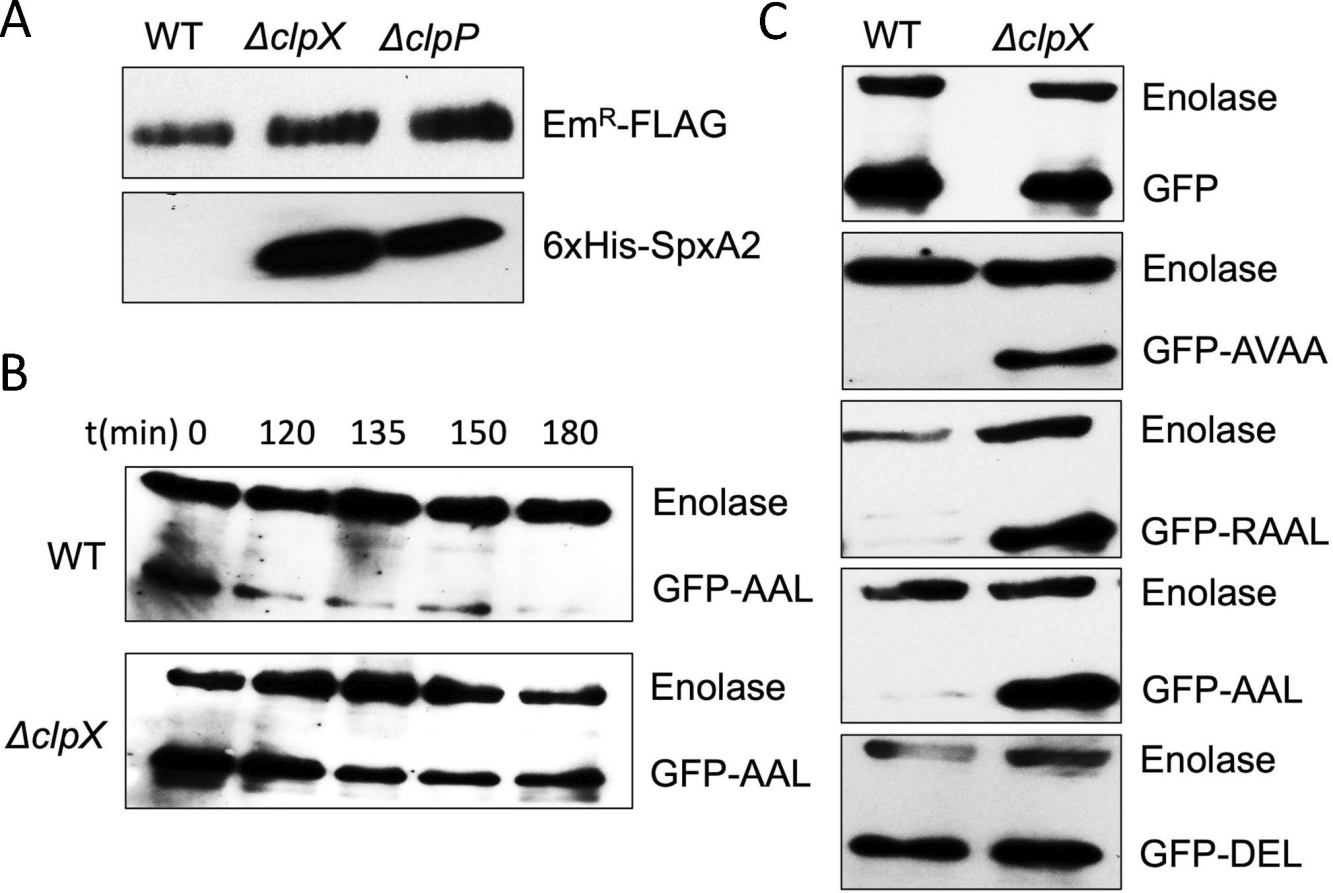
SpxA2 is a ClpX/P substrate, and its C-terminal alanine residues are critical for its degradation. (**A**) Western blot analysis of SpxA2 expressed with a His-tag at its N-terminal using pIBY35 shuttle vector in the wild-type (UA159) and its isogenic *ΔclpX* (IBSJ4) and *ΔclpP* (IBS512) strains. Erythromycin-resistant methylase with a FLAG-tag at its C-terminus (Em^R^-FLAG) encoded by pIBY35 served as a loading control. (**B**) Western blot analysis of GFP-AAL degradation after adding chloramphenicol (20 µg/mL) to the culture at OD_600_ ∼0.6 and incubated further as indicated. Enolase was used as a loading control. Experiments were performed at least two times, and representative gels are shown. (**C**) Western blot analysis of GFP, GFP-AVAA, GFP-RAAL, GFP-AAL, and GFP-DEL expressed in the wild-type and the *ΔclpX* strains using the pIB190 shuttle vector. GFP and GFP-AVAA served as negative and positive controls, respectively. Enolase was used as a loading control.

The second most differentially accumulated protein in the *ΔclpX* strain compared to the wild-type strain was SpxA2, a transcriptional regulator (5.6-fold) (Table 1). Since we previously demonstrated that SMU.961, which is among the top five differentially accumulated proteins (4.6-fold), is an authentic ClpX substrate, we predicted that SpxA2 is a ClpX substrate as well (22, 39). It is also noteworthy to mention that SpxA2 is a well-characterized ClpX substrate in many Gram-positive bacteria. To verify whether ClpX can recognize SpxA2, we cloned the *smu.2084 (spxA2*) gene in a shuttle expression vector pIBY35 under a constitutive P_23_ promoter to generate pIBW43. Plasmid pIBW43 constitutively expresses SpxA2 with a N-terminal His-tag, leaving the C-terminal part of the protein unaltered. The plasmid was then introduced into IBSJ4 (*ΔclpX*), IBS512 (*ΔclpP*), and UA159 (wild type) strains by natural transformation. The strains were grown in nutrient-rich THY broth at 37°C to stationary phase, and the cellular proteins were extracted. We then checked the abundance of SpxA2 by Western blotting using an anti-His antibody. As shown in Fig. 1A, we observed that SpxA2 was not detected in the wild-type strain, whereas the protein was detected at an increased level in both the *ΔclpX* and *ΔclpP* strains. We also observed that the amount of accumulation in *ΔclpX* and *ΔclpP* strains was comparable. This result indicates that ClpX/P is indeed involved in the recognition and degradation of SpxA2 protein in the *S. mutans* UA159 strain.

### C-terminal amino acids of SpxA2 are important for its ClpX/P-mediated degradation

Generally, ClpX recognizes short motif sequences at the N- or C-terminal end of the substrates (10). We have previously found that the short sequences at the C-terminus of substrates are crucial for ClpX/P-mediated degradation in *S. mutans* (22, 32). When we scanned the C-terminal region of SpxA2, we noticed a tripeptide sequence containing alanine and leucine residues (AAL) with a high resemblance to the SsrA tag sequence (AVAA) present. This AVAA sequence is a *bona fide* ClpX degradation signal in *S. mutans* (40). The pIBY35 shuttle vector generates N-terminal His-tagged proteins. Thus, we fused 3 to 10 residues of the C-terminal SpxA2 to the C-terminus of a GFP (Table 2). We then inhibited the *de novo* protein synthesis in the wild-type and *ΔclpX* strains containing plasmids by adding chloramphenicol to the culture and checked for the accumulation of GFP-AAL protein. As shown in Fig. 1B, we observed that the accumulation of GFP-AAL was reduced in both the wild-type and the *ΔclpX* strains. However, the reduction in GFP-AAL in the Δ*clpX* strain was much slower than in the wild-type strain. These results suggest that the tri-peptide sequence “AAL” at the C-terminal of SpxA2 was sufficient for the ClpX-dependent degradation of GFP (Fig. 1B) in *S. mutans*, and GFP can be used to evaluate degradation signal at the C-terminal of ClpX substrates.

**TABLE 2:** Degradation pattern of the GFP tagged with variants of C-terminal amino acids from SpxA2^*a*^

GFP-X	WT (UA159)	*ΔclpX* (IBSJ4)
-NTAARLRAAL	^+^	−
-RAAL	^+^	−
-EAAL	^+^	−
-AAL	^+^	−
-AAV	^+^	−
-AAI	^+^	−
-AIL	^+^	−
-VAL	^+^	−
-AVL	^+^	−
-ADL	−	−
-DEL	−	−

^*a*^ + denotes degradation of GFP fusion protein; – denotes non-degradation of GFP fusion. Accumulation of >80% GFP fusion proteins with respect to GFP protein alone (without tag) is considered non-degradation (–).

Generally, the degradation motifs contain highly conserved residues, and replacing those conserved residues generates motifs that are not recognized by ClpX/P (22, 32, 41, 42). Therefore, we further created variants of the AAL motif to identify the key amino acid residues. All such constructs are listed in Table 2. Based on the physicochemical properties of the residues, we replaced each residue with valine (V). We also substituted some of the residues with isoleucine (I). We found that substituting A or L residues with V, I, or L residues did not affect the ClpX/P-mediated degradation (Table 2). Furthermore, as a specificity control, we replaced the A residue with a negatively charged residue such as aspartate (D) and glutamate (E) and observed that substituting hydrophobic residues with charged ones led to loss of GFP fusion protein degradation (Fig. 1C; Table 2). As expected, the positive control construct, GFP-AVAA, also degraded in the wild-type strain but not in the *ΔclpX* UA159 strain (Fig. 1C). Furthermore, the fourth residue, arginine (R) from the C-terminal of Spx2, if replaced by glutamate (E), did not interfere with degradation. These results suggest that any hydrophobic residue in the tripeptide motif at the C-terminal of a substrate serves efficiently as a degradation signal.

### Identification and evaluation of additional ClpX/P degradation motifs in *S. mutans*

**Fig 2:**
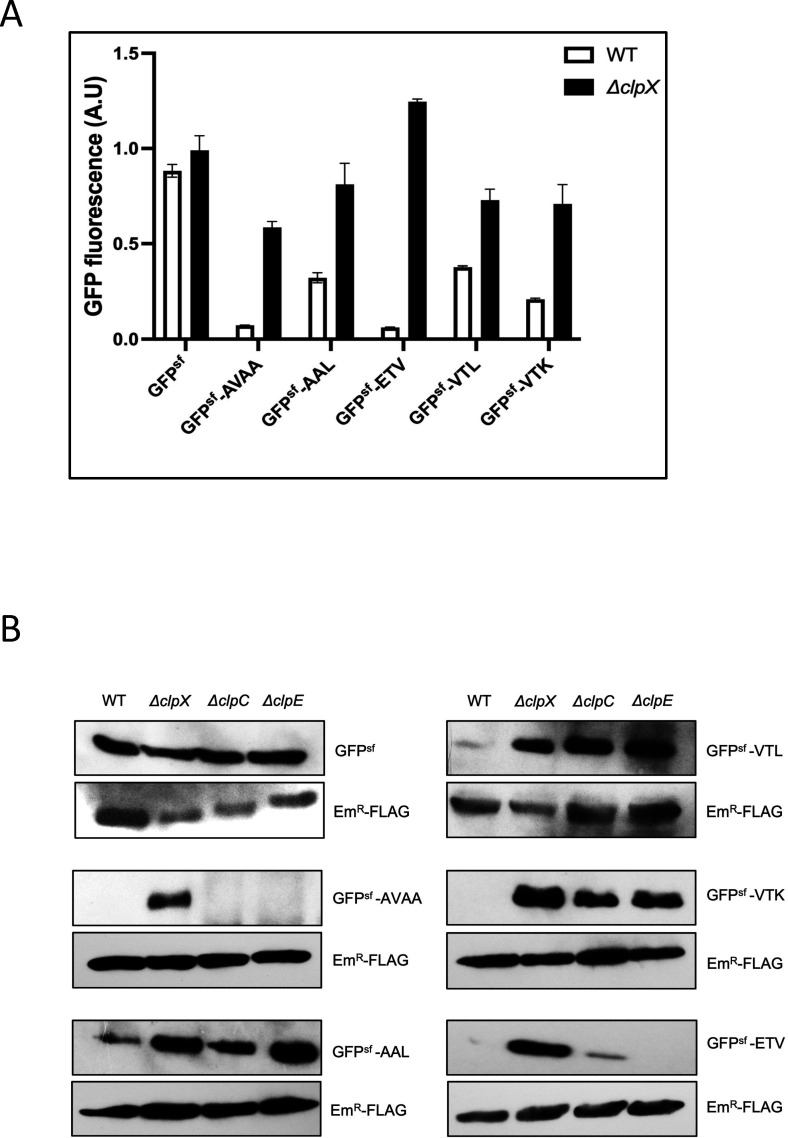
ClpX degradation motifs in *S. mutans*. (**A**) C-terminal amino acids of SMU.932, SMU.933, and SMU.962 are ClpX substrates. GFP fluorescence measurement of the wild-type and the *ΔclpX* strains carrying GFP^sf^-AAL, GFP^sf^-ELQ, GFP^sf^-ETV, GFP^sf^-VTL, and GFP^sf^-VTK constructs. Cells were harvested at late stationary phase, and fluorescent was measured as described in Materials and Methods. GFP^sf^-AVAA construct was served as positive control, and GFP^sf^ was as a negative control. The GFP^sf^ fluorescence value of GFP^sf^ in the *clpX*-deleted strain was set as 1. Experiments were performed in triplicates and repeated at least twice. The error bar represents mean ± SD. (**B**) ClpC and ClpE both recognize AAL, VTL, and VTK motifs for degradation in *S. mutans*. Western blot assays of the constructs carrying GFP^sf^, GFP^sf^-AAL, GFP^sf^-VTL, GFP^sf^-VTK, and GFP^sf^-ETV fusions were expressed in the wild-type (UA159) and its isogenic *ΔclpX* (IBSJ4), *ΔclpC* (IBSJ2), and *ΔclpE* (IBSJ5) strains using pIBY35 shuttle vector. FLAG-tagged erythromycin-resistant methylase (Em^R^-FLAG) encoded by the vector was used as a loading control. Experiments were performed at least two times, and representative gels are shown.

Our proteomic data revealed that several proteins (~11) accumulated >2.0-fold in the Δ*clpX* strain. Among them, SsrA (tm-RNA), SMU.961, SMU.932, SMU.933, and SMU.962 were identified as the top putative substrates (Table 1). We have previously reported that SMU.961 is an authentic ClpX/P substrate and identified “ELQ” tripeptide as a ClpX/P degradation motif (22). We wondered whether the tripeptide motifs present at the ultimate C-terminal end of SMU.932, SMU.933, and SMU.962 (Fig. S1) would function as degradation signals for ClpX/P. To this end, we employed a super-folder GPF (GFP^sf^) that is easily monitored *in vivo* instead of a cumbersome Western blot assay. In this *in vivo* assay, we first verified that the negative control (GFP^sf^) and the positive control (GFP^sf^-AVAA) controls behave as expected (Fig. 2A). We then constructed fusions with the last three residues of the SMU.962 (ETV), SMU.933 (VTL), and SMU.932 (VTK) tagged with GFP^sf^. When we measured the fluorescence of GFP^sf^-ETV, GFP^sf^-VTL, and GFP^sf^-VTK in the wild-type and the *ΔclpX* strains, we found that the fluorescence for each of the above GFP^sf^ fusion constructs was higher in the *ΔclpX* strain as compared to the wild-type strain (Fig. 2A). Taken together, our results suggest that ETV, VTL, and VTK motifs function as ClpX/P degradation signals in *S. mutans*. However, the loss of fluorescence is not as strong for AAL, VTL, and VTK as for AVAA and ETV.

Since some ClpX degradation motifs are often recognized by other ClpP interacting Clp ATPases such as ClpA, ClpC, or ClpE (40, 43, 44) (22, 40), therefore, we verified whether ClpC and ClpE could also recognize these new degradation motifs. To this end, to further confirm our *in vivo* results (Fig. 2A), we performed traditional Western blot assay and found that they correlate with the *in vivo* degradation observation (Fig. 2B), and we checked the accumulation of GFP^sf^-AAL, GFP^sf^-ETV, GFP^sf^-VTL, and GFP^sf^-VTK fusion proteins by Western blot assay in the wild-type, *ΔclpC*, and *ΔclpE* strains. As shown in Fig. 2B, we observed that degradation of GFP^sf^-AAL, GFP^sf^-VTL, and GFP^sf^-VTK also depends on both ClpC and ClpE ATPases. These results strongly indicate that AAL, VTL, and VTK motifs also serve as degradation signals for ClpC and ClpE. However, we observed that GFP^sf^-ETV fusion was degraded in the *ΔclpE* strain, and the accumulation of fusion protein was greatly reduced in the *ΔclpC* strain (Fig. 2B). Thus, it suggests that GFP^sf^-ETV is not dependent on ClpE/P for its degradation but is marginally dependent on ClpC/P.

### *In vitro* degradation of the motifs by ClpX/P

**Fig 3:**
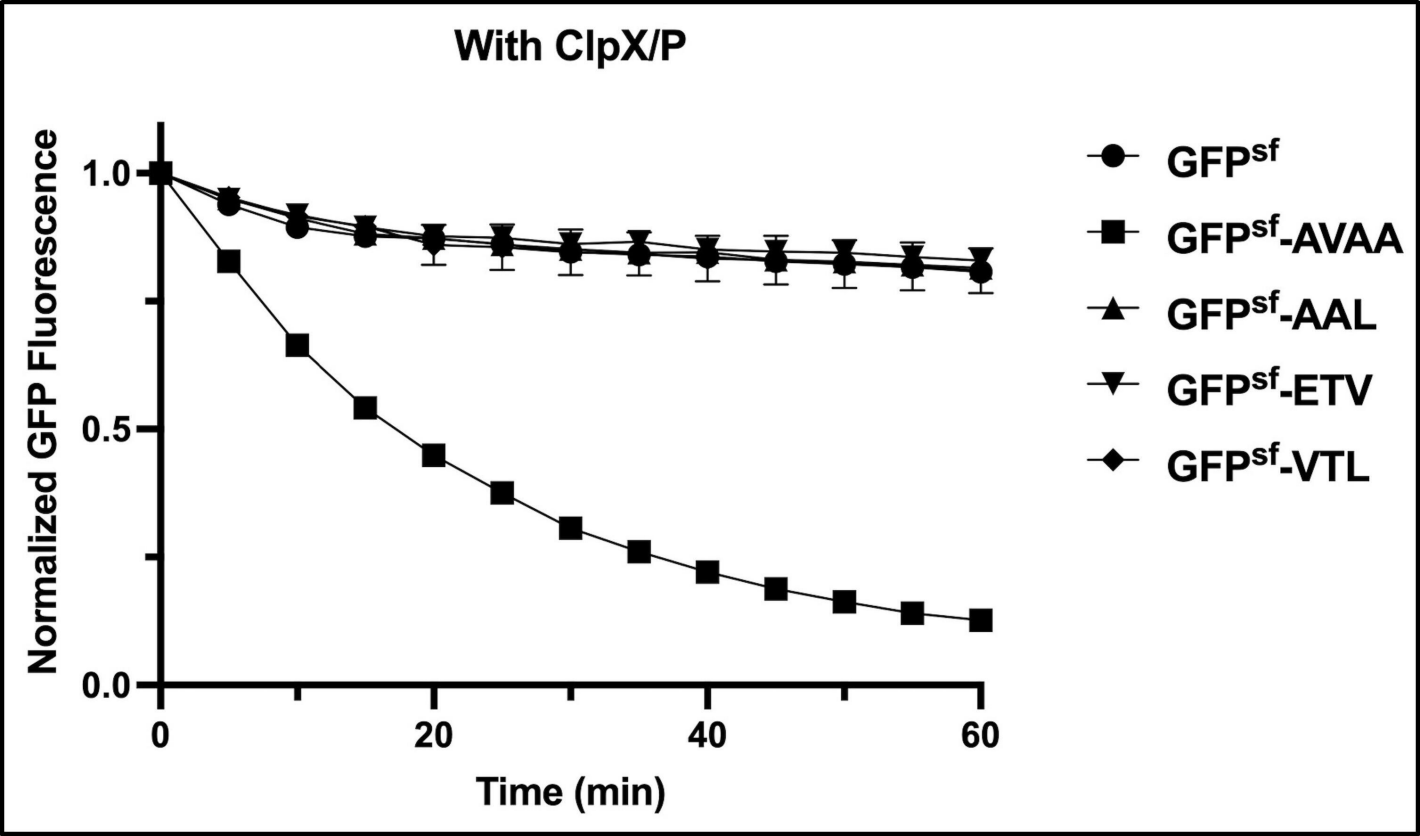
*In vitro* degradation assay of various motifs by ClpX/P. Purified GFP tagged substrates (GFP^sf^, GFP^sf^-AVAA, GFP^sf^-AAL, GFP^sf^-ETV, and GFP^sf^-VTL) were incubated with preformed ClpX/P complex proteins for fluorescent measurement. The details of the degradation assay setup are described in Materials and Methods. GFP fluorescence (λ_exi_ = 467 nm and λ_emi_ = 511 nm) was measured at various time points as indicated, and the initial fluorescence value was set as 1. All the experiments were performed in triplicates. The error bar represents mean ± SD.

ClpX/P recognizes its substrate directly or with the aid of additional proteins called adaptor proteins (15). Several studies, including ours, have shown that many ClpX/P substrates that are degraded *in vivo* remain undegraded in an *in vitro* degradation assay (22, 32, 39). Therefore, we wanted to evaluate whether ClpX/P can efficiently degrade the newly identified motifs *in vitro*. We used purified ClpX, ClpP, and the substrates GFP^sf^-AAL, GFP^sf^-VTL, and GFP^sf^-ETV. We also used purified GFP^sf^ and GFP^sf^-AVAA proteins as negative and positive controls, respectively. As expected, purified ClpP or ClpX proteins alone did not show any degradation of the positive control GFP^sf^-AVAA (Fig. S2); however, reconstituted ClpX/P complex efficiently degraded GFP^sf^-AVAA as the GFP^sf^ fluorescence was rapidly decreased over time in the presence of both ClpX and ClpP proteins. In contrast, we found that the reconstituted ClpX/P complex was not able to degrade any of the other fusion substrates as the fluorescence remained unchanged over time (Fig. 3). Furthermore, the addition of crude cell lysate prepared from the stationary phase culture to the reconstituted ClpX/P did not result in any change in fluorescence (data not shown). These data suggest that AAL, VTL, or ETV motifs are not degraded *in vitro* assay either due to suboptimal conditions or due to the absence of an accessory factor or metabolite.

### Recognition of AAL, ETV, and VTL by ClpX is dependent on its zinc-binding domain

**Fig 4:**
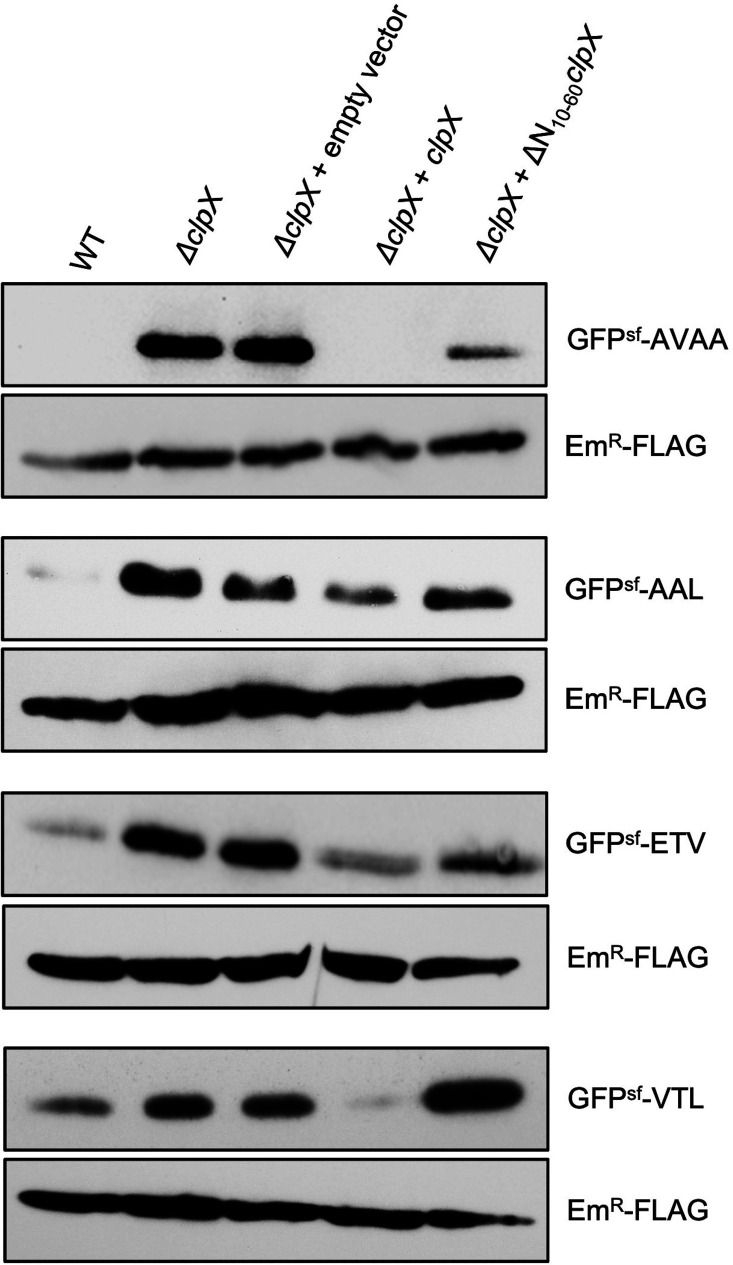
Role of ZBD of ClpX in motif recognition. The *ΔclpX* mutant strain IBSJ4 was complemented with pIB166 vector carrying full-length *clpX* (pIB1F13) or ZDB-deleted *clpX* (ΔN_10-60_ *clpX*; pIB1F24). In these strains, vector pIBY35 expressing GFP^sf^ variants were introduced. Whole cell lysates were prepared from stationary phase cultures, separated by SDS-PAGE in duplicate, and subjected to Western blot analyses. One blot was probed with rabbit anti-GFP antibody, and the other blot was probed with rabbit anti-FLAG antibody. Both blots were then probed with goat anti-rabbit HRP-conjugated secondary antibody and developed with ECL chemiluminescent kit. The blots were exposed to X-ray film and then photographed. The experiment was repeated at least twice, and a representative blot is shown.

The ZBD of ClpX plays a crucial role in dimerization and substrate recognition in *E. coli* and other bacteria (18). However, its function in Gram-positive bacteria, including streptococci, is largely unknown. Studies suggest that adaptor proteins bind to the ZBD and deliver substrates to ClpX/P for degradation (19, 45, 46). Since we observed that ClpX/P did not degrade the GFP^sf^ fusion proteins *in vitro*, we speculated that adaptor proteins exist which recognize the proteins with the newly identified degradation motifs. Thus, we used a complementation system to evaluate the function of ZBD in the recognition of motifs by ClpX that we identified. The *ΔclpX* mutant strain was complemented with either a full-length (pIB1F13) or a ZBD-deleted ClpX (pIB1F24) constructs. Growth verification assay demonstrates that both complemented strains have similar growth characteristics (Fig. S3). In these strains, we also introduced the GFP^sf^-AAL, GFP^sf^-ETV, and GFP^sf^-VTL fusion constructs and performed Western blot analysis. As shown in Fig. 4, we observed that GFP^sf^-AAL, GFP^sf^-ETV, and GFP^sf^-VTL fusion proteins accumulated in the *ΔclpX* and *ΔclpX* complemented with the empty vector (pIB166) or with ZBD-deleted ClpX construct (pIB1F24). In contrast, when we used the full-length ClpX construct (pIB1F13) for complementation, we observed little to no accumulation of GFP^sf^ fusion proteins. The accumulation pattern was similar to the accumulation pattern in the wild-type strain. These results strongly suggest that the ZBD of ClpX is involved in recognizing AAL, ETV, and VTL motifs in *S. mutans.*

## DISCUSSION

In *S. mutans*, ClpX/P complex is the major intracellular protease involved in maintaining protein homeostasis in the cell. While the amount of the proteolytic component ClpP is inducible by various cellular stresses, the amount of the ATPase component, ClpX, is relatively stable throughout the growth (12). Consistent with ClpX/P’s key role in regulated proteolysis, inactivation, or functional loss of either ClpX or ClpP leads to pleiotropic effects, including growth defects, aberrant biofilm formation, reduced bacteriocin production, improper natural competence development, and others physiological effects (31, 47, 48). While the importance of ClpX/P in cellular physiology is unraveling, the identity of the substrates targeted by this proteolytic complex is largely unknown in *S. mutans* and other streptococci. Our research group has attempted to identify the ClpX substrates using directed or proteomic approaches such as a two-dimensional gel electrophoresis (22, 32). Those studies have identified two motif sequences (LPF and ELQ) that act as degradation/recognition signals for ClpX in *S. mutans.* To expand our knowledge about the identity of the substrates recognized by ClpX under non-stressed conditions, we used a TMT-based proteomics approach. To maximize our search, we set a cut-off value of 1.2-fold, since our previous proteomic studies have found that the proteins whose accumulation is deviated 20% or greater from the control baseline are generally authentic substrates (22, 32, 35, 49). At this cut-off value, we found that nearly 15% of the *S. mutans* proteome is modulated by ClpX (Table S3). While this value seems a little higher as compared to the other studies, we believe it represents a comprehensive picture of the cellular proteome status that might be affected either directly or indirectly by ClpX (50).

We believe the proteins whose accumulation were upregulated the most in the *clpX* mutant (~8.5% of the proteome) are authentic ClpX substrates. When we tested a subset of them, indeed, we found all of them were degraded by ClpX/P. The most upregulated substrate was SpxA2, a transcriptional regulator involved in stress tolerance response in bacteria (51). Previously, Ganguly and colleagues have also found that SpxA2 is a *bona fide* substrate of ClpX/P in *S. mutans* (39). Our study suggested that the terminal AAL residues of SpxA2 were important for ClpX-mediated recognition and degradation (Table 2; Fig. 2). Since this motif contains only hydrophobic residues, we substituted this tripeptide motif with other hydrophobic residues, such as valine (V) or isoleucine (I) (Table 2). When we substituted the motif with charged residues such as aspartic acid (D) or glutamic acid (E), ClpX was unable to degrade (Table 2). We previously found that LPF and the AVAA (SsrA) motifs are authentic ClpX degradation motifs (32, 40). This suggests that ClpX has some preference for motifs that are hydrophobic in nature.

It is noteworthy that when we looked *in silico* for substrates with terminal AAL motif in the *S. mutans* UA159 genome, we only found SpxA2 and not any other proteins. In *Caulobacter crescentus*, McpA, a transmembrane chemoreceptor, is degraded in a ClpX/P-dependent manner. The AAL motif, crucial for degradation, is located 15 residues away from the C-terminus (**AAL**AQAPASDGWEEF). Sequential deletions show that the 12 residues after AAL motif are not essential for McpA degradation (52). When we searched the *S. mutans* UA159 genome, we found about 10 proteins where AAL motif is located near the C-terminus (Table 3). While we have not tested any of these proteins for ClpX-mediated degradation, some could be degraded by ClpX/P in *S. mutans*.

**TABLE 3:** *S. mutans* UA159 proteins with the tri-peptide sequence AAL located near the C-terminal end and the tri-peptide sequences VTL, VTK, and ETV at the ultimate C-terminal end

Locus tag	Protein description	AAL, VTL, VTK, and ETV location at the C-terminal sequence
SMU.352	Putative ribulose-phosphate-3-epimerase	AALND
SMU.361	Phosphoglycerate kinase	AALTEK
SMU.795	Conserved hypothetical protein	AALSQ
SMU.924	Thiol peroxidase	AALAAVK
SMU.929	Conserved hypothetical protein	AALLYLF
SMU.1006	Putative ABC transporter, ATP-binding protein	AALLGGE
SMU.1117	NADH oxidase (H_2_O forming)	AALKAK
SMU.1191	6-Phosphofructokinase	AALNRDLSN
SMU.1675	Putative cystathionine gamma-synthase	AALEA
SMU.2031	Putative translation elongation factor TS	AALNK
SMU.1037	Putative histidine kinase	VTL
SMU.1718	Putative glutamate racemase	VTL
SMU.933	Putative ABC transporter	VTK
SMU.572	Putative tetrahydrofolate dehydrogenase	VTK
SMU.440	Hypothetical protein	ETV
SMU.811	Hypothetical protein	ETV
SMU.962	Putative dehydrogenase	ETV

SpxA2 is a known substrate for ClpX/P-mediated degradation in many Gram-positive bacteria; however, the degradation motif was not known (39). SpxA2 is a highly conserved protein among Gram-positive bacteria. However, when we analyzed *in silico* the C-terminal motif of SpxA2, we found that the motif varies significantly among other bacterial species. For *B. subtilis*, the C-terminal motif for SpxA2 appears to be RLAN, whereas for *L. monocytogenes* and *S. aureus*, the motif appears to be KMVN and RMVD. On the other hand, most of the streptococcal SpxA2 encode RAAL motif. However, we also found that some streptococci encode other variants at the C-terminus of SpxA2 (such as RAAF, RAVL, RGAL, RSTL, RTAL, and others). Whether these motifs are recognized by ClpX/P in the respective organisms needed to be experimentally verified.

Some Gram-positive bacteria including streptococci encode two SpxA homologs, SpxA1 and SpxA2. While SpxA1 is a ClpX substrate in many Gram-positive bacteria, we found that the C-terminus region of SpxA1 encodes GEED motif, which is not a recognition motif for ClpX/P. The GEED motif appeared to be highly conserved among streptococci; thus, we believe that SpxA1 is not a substrate for ClpX/P at least in this genus. Moreover, previously, Ganguly and colleagues (39) demonstrated that SpxA2, not SpxA1 is subjected to ClpXP proteolysis.

With a similar approach, we also determined the motifs recognized by ClpX and found that the last three residues are crucial for ClpX/P-mediated degradation (Table 2; Fig. 2). These motifs, ETV, VTL, and VTK, are somewhat different from the purely hydrophobic residue-containing motifs such as AAL or LPF. However, they all contain at least one hydrophobic residue. At present, we do not know which residues in these motifs are critical for ClpX-dependent degradation, as we have not done substitution studies like the AAL motif. When we searched the *S. mutans* UA159 genome for the presence of these motifs, we identified several additional proteins containing ETV, VTL, or VTK motifs (Table 3). However, these additional proteins were not identified in our proteomic assay (Table S3). One possibility is that the abundance or the expression of these proteins is very low in the cell or expressed only under certain growth conditions. Nevertheless, since the tripeptide motifs alone are recognized *in vivo* by ClpX/P (Fig. 2), we strongly believe that these additional proteins are also ClpX/P substrates.

We were unable to demonstrate the degradation of the identified motifs in *in vitro* assay using purified reconstituted ClpX/P complex. There could be several reasons for this unexpected observation. First, additional factors such as adaptor proteins or metabolites may be needed for *in vitro* degradation. Second, the motifs could be post-translationally modified *in vivo* to be recognized by ClpX/P, which is absent in our *in vitro* studies, since the substrate proteins were purified from *E. coli*. Another possibility is that GFP is folded differently in *S. mutans* than in *E. coli*. As a result, GFP fusion constructs are not recognized in *in vitro* assays. Further in-depth studies are needed to understand the process thoroughly.

Among the various Clp ATPases, ClpX is the only one that contains a single AAA+ ATPase domain; all other contain two AAA+ domains. The role of this single ATPase domain is crucial for substrate unfolding and threading to the proteolytic ClpP chamber. The other domain that is important for the function of ClpX is the ZBD. ClpE ATPase also contains a ZBD but not ClpC (6). We found that the ZBD of ClpX has a differential role in substrate recognition and degradation. For AAL, ETV, and VTL motifs, ZBD is necessary for degradation as deletion of this domain led to failure in degradation (Fig. 4). For the AVAA tag degradation, it seems that when ZBD was absent, some degradation was observed (Fig. 4). In *E. coli*, an adaptor protein, RssB, recognizes the substrate sigma S and delivers to ClpX/P complex for degradation by interacting with the ZBD of ClpX (19). On the other hand, ZBD is not required for GFP-SsrA degradation in *E. coli* (18). However, an adaptor protein, SspB, enhances the degradation rate of SsrA tagged substrates by interacting with the ZBD (45, 46). Our results indicate that SsrA-tag is degraded in the absence of ZBD, although not as efficiently as when the ZBD is present.

Some of the motifs that we identified here are also recognized by other Clp ATPases. For example, AAL, VTK, and VTL are also recognized by ClpC and ClpE, as these substrates accumulated when the respective ATPases were absent. In contrast, the ETV motif is predominantly degraded by ClpX and, to some extent, by ClpC (Fig. 2). It is important to mention that we previously found that LPF motif was also not recognized by ClpC or ClpE (32), but only by ClpX. While the exact reasons for this differential motif recognition are not currently understood, it is possible that these motifs need redundant mechanisms for their recognition depending on the cellular physiology. While the major role of ClpX is maintaining the cellular proteome, the role of ClpC and ClpE is during stress response. ClpC and ClpE ATPases are induced under thermal and other stresses, while ClpX is not (12). Thus, we speculate that for certain substrates, multiple Clp ATPases are necessary for degradation.

It is somewhat surprising to find that some of these motifs such as AAL, VTK, and VTL have been accumulated in the single mutant where other two ATPases are present. For example, we found accumulation in the ClpC mutant cells, where both the ClpX and ClpE ATPases are active (Fig. 2). We believe that the degradation for these motifs by different Clp ATPases requires different factors such as adaptor proteins that are differentially modulated by different Clp ATPases. It is also possible that degradation of a given substrate might be accumulative. To understand the contribution of each of the Clp ATPase, one needs to study the accumulation of these substrates in the double and triple mutant cells and to compare with the accumulation in ClpP inactivated strain, which is beyond the scope of the current study.

In this study, we identified the putative substrates for ClpX/P proteolytic complex and used a TMT-based proteomic assay. We also developed an *in vivo* assay using sf-GFP to confirm the motifs that are recognized by ClpX/P. While this strategy is straightforward and undoubtedly identifies novel degradation/recognition motifs, it is somewhat laborious. The other alternative methods, such as the use of a trap mutant that cannot hydrolyze ATP and thus “traps” the substrate or the use of membrane immobilized peptide arrays, also suffer drawbacks (53, 54). However, we believe that the recent technical advancement of peptide array synthesis, such as SPOT synthesis coupled with *in situ* direct binding with either ClpX alone or ClpX/P in the presence of non-hydrolyzable ATP, would be an attractive method, which we are considering for our future study (55 – 57).

## SUPPLEMENTAL MATERIAL

Supplemental material spectrum.03457-23-s0001.pdf Figures S1 to S3 and Tables S1 to S3.

Table S4 spectrum.03457-23-s0002.xlsx TMT raw data.

ASM does not own the copyrights to Supplemental Material that may be linked to, or accessed through, an article. The authors have granted ASM a non-exclusive, world-wide license to publish the Supplemental Material files. Please contact the corresponding author directly for reuse.
