# Supplementary material for: Diverse nature of ClpX degradation motifs in Streptococcus mutans
Source: Microbiol Spectr. 2023 Dec 5;12(1):e03457-23. doi: 10.1128/spectrum.03457-23 (PMC10782952; doi:10.1128/spectrum.03457-23)
Supplement: Supplemental material — Figures S1 to S3 and Tables S1 to S3. [file spectrum.03457-23-s0001.pdf]

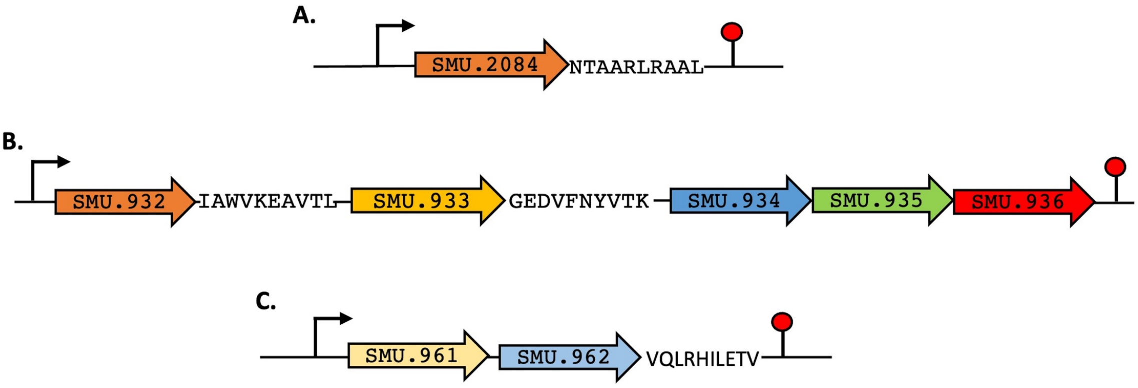

Fig S1. Genomic arrangement of (A) *smu.2084*, (B) *smu.932 and smu.933* (C) *smu.962* with their C-terminal amino acid sequences in UA159.

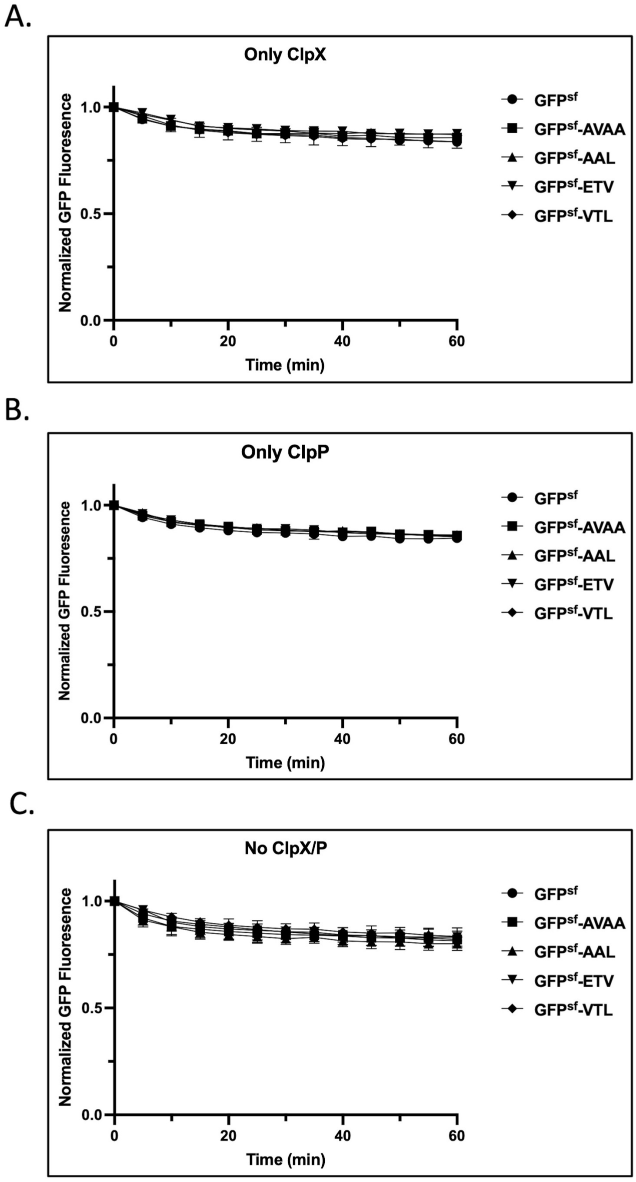

Figure S2. *In vitro* degradation of GFP-AAL, GFP-ETV, GFP-VTL and GFP-VTK by ClpX alone, ClpP alone and in absence of both ClpX and ClpP. 0.1μM of purified GFP^{sf} -AAL, GFP^{sf} –ETV, and GFP^{sf}-VTL proteins were incubated with (A) ClpX (1μM), (B) ClpP (1.2μM) and (C) neither ClpX or ClpP in a ClpX/P degradation assay setup as described in materials and methods. GFP-fluorescence was measured at λ_{exi} = 467nm and λ_{emi} = 511nm, and the initial GFP fluorescence value was set as 1. Purified GFP^{sf}-AVAA and GFP^{sf} proteins served as positive and negative controls respectively. All experiments were performed in triplicates.

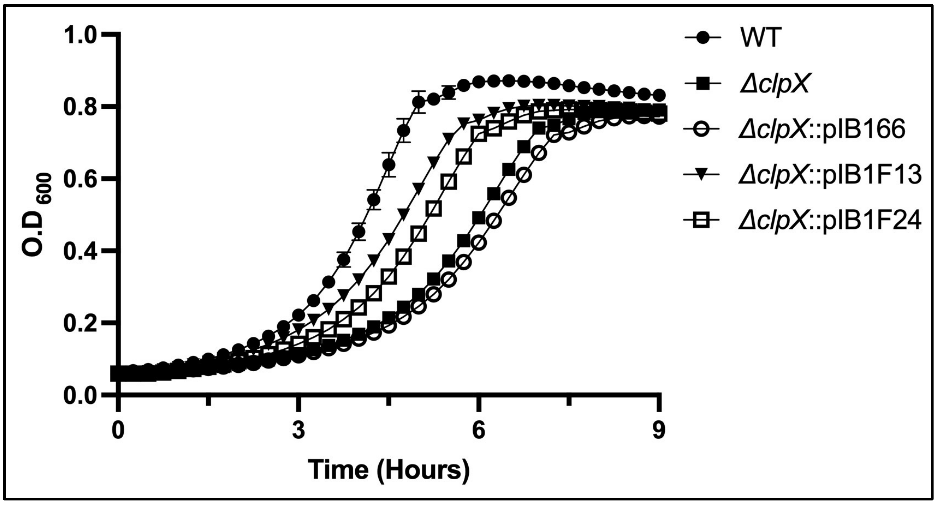

Figure S3. Growth kinetics of *S. mutans* UA159 and its derivatives transformed with pIB1F18 (pIBY35::*gfp^sf^-avaa*). UA159 derivative strains (wild type, *ΔclpX*, *ΔclpX*::pIB166, *ΔclpX*::pIB1F13 and *ΔclpX*::pIB1F24) expressing GFP^{sf}-AVAA were grown in THY medium at 37°C under microaerophilic conditions. *ΔclpX* was complemented by expressing the full-length ClpX (*ΔclpX*::pIB1F13), and the zinc-binding domain deleted ClpX (*ΔclpX*::pIB1F24) under the control of a constitutive P_{23} promoter from a shuttle vector pIB166 and containing empty pIB166 as a vector control. The optical densities of the cultures at 600 nm were recorded for 9 hours using a microtiter plate reader (Biotek). All experiments were done in triplicates. The growth curves shown are the averages with the standard deviations.

Table: S1. Differentially expressed proteins in *S. mutans* Δ*clpX* strain

Accession #	Description	**Score**	**Δ*clpX*/WT**	MS2 scans	peptides	emPAI
SMU.RS05510	tmRNA (SsrA-tag)	79	7.034	3	1	2.8
SMU_2084	Transcriptional regulator SpxA2	192	5.619	8	3	0.9
SMU_962	Putative dehydrogenase	109	4.93	4	3	0.23
SMU_961	Conserved hypothetical protein	188	4.64	15	6	1.23
SMU_932	Hypothetical protein	175	3.794	11	3	0.23
SMU_116	Tagatose 1,6-aldolase	28	2.51	1	1	0.07
SMU_933	Putative amino acid ABC transporter	50	2.281	4	3	0.24
SMU_310	Sorbitol operon activator	16	2.112	1	1	0.14
SMU_1245	Conserved hypothetical protein	51	2.096	2	1	0.13
SMU.2074	Ribonucleoside-triphosphate reductase	1027	2.054	45	12	0.6
SMU_1722	Putative integral membrane protein	42	2.041	1	1	0.12
SMU_117	Conserved hypothetical protein	30	1.994	1	1	0.06
SMU_837	Putative reductase	598	1.958	31	6	0.93
SMU_1438	Putative Zn-dependent protease	85	1.867	4	2	0.22
SMU_118	Putative esterase	61	1.822	2	1	0.18
SMU_274	Putative hexulose-6-P isomerase	111	1.809	4	1	0.08
SMU_1003	Putative division protein	144	1.79	4	2	0.11
SMU_1723	Conserved hypothetical protein	51	1.787	3	1	0.13
SMU_308	Sorbitol-6-phosphate 2-dehydrogenase	195	1.768	7	2	0.2
SMU_1322	Acetoin dehydrogenase	113	1.757	17	2	0.34
SMU_1296	Glutathione S-transferase	675	1.726	28	7	1.41
SMU_1265	Ribonucleotide isomerase	49	1.713	1	1	0.11
SMU_272	PTS system, enzyme IIA component	48	1.712	1	1	0.16
SMU_273	Hexulose-6-phosphate synthase	221	1.696	12	3	0.38
SMU_267	Glutamate-cysteine ligase	239	1.679	13	5	0.17
SMU_2028	Beta-D-fructosyltransferase	547	1.655	25	11	0.44
SMU_618	Hypothetical protein	60	1.655	2	1	0.28
SMU_1945	Hypothetical protein	243	1.654	13	5	0.51
SMU_112	Putative transcriptional regulator	21	1.643	1	1	0.09
SMU_180	Putative oxidoreductase	330	1.635	15	6	0.21
SMU_275	L-ribulose 5-phosphate 4-epimerase	144	1.635	7	4	0.64
SMU_1004	Glucosyltransferase-I	5730	1.608	236	35	1.38
SMU_32	PRPP amidotransferase (GPAT)	77	1.58	4	2	0.11
SMU_677	Transcriptional regulator (MerR family)	164	1.565	3	1	0.19
SMU_448	Hypothetical protein	136	1.564	8	4	0.93
SMU_527	Conserved ypothetical protein	60	1.564	2	1	0.07
SMU_1436	Hypothetical protein	41	1.563	2	1	0.09
SMU_603	Putative D-Ala-D-Ala ligase	34	1.558	1	1	0.05
SMU_669	Putative glutaredoxin	45	1.549	3	1	0.3
SMU_888	UDP-galactose 4-epimerase, GalE	362	1.546	16	5	0.51
SMU_1432	Putative endoglucanase precursor	58	1.514	3	2	0.15
SMU_799	Conserved hypothetical protein	26	1.512	1	1	0.21
SMU_1679	Conserved hypothetical protein	34	1.499	3	1	0.14
SMU_119	Putative alcohol dehydrogenase	136	1.487	9	3	0.23
SMU_383	Conserved hypothetical protein	38	1.482	3	2	0.14
SMU_312	Sorbitol phosphotransferase (IIBC)	169	1.478	5	2	0.16
SMU_540	Peroxide resistance protein Dpr	248	1.477	18	4	0.71
SMU_205	hypothetical protein	51	1.477	2	2	0.56
SMU_992	hypothetical protein	17	1.476	1	1	0.07
SMU_1268	Glycerol-phosphate dehydratase	44	1.474	2	1	0.13
SMU_689	Hypothetical protein	758	1.473	43	17	0.67
SMU_1787	Putative secreted protein	450	1.469	19	3	0.7
SMU_1215	Uracil DNA glycosylase	159	1.469	4	1	0.24

SMU_1273	Histidinol-phosphate aminotransferase	321	1.464	19	8	0.71
SMU_678	Putative oxidoreductase	252	1.462	14	3	0.28
SMU_1442	Conserved hypothetical protein	694	1.461	47	7	1.43
SMU_1270	Histidinol dehydrogenase	389	1.46	24	8	0.59
SMU_210	Hypothetical protein	37	1.46	2	2	0.52
SMU_167	Hypothetical protein	47	1.459	4	2	0.35
SMU_1005	Glucosyltransferase-SI	5903	1.456	260	36	1.45
SMU_1412	Putative ABC transporter	505	1.455	28	10	0.35
SMU_1203	Branched-chain aminotransferase	297	1.435	11	5	0.53
SMU_1681	Conserved hypothetical protein	674	1.434	17	3	1.14
SMU_1423	Putative pyruvate dehydrogenase	131	1.431	12	5	0.49
SMU_679	putative oxidoreductase	96	1.426	4	3	0.28
SMU_1867	Putative alcohol dehydrogenase	517	1.421	15	5	0.43
SMU_179	Conserved hypothetical protein	327	1.411	16	3	0.62
SMU_1098	Putative oxidoreductase	208	1.391	9	4	0.33
SMU_1534	ATP synthase subunit c	31	1.386	1	1	0.42
SMU_34	Phosphoribosyl imidazole synthetase	191	1.384	9	5	0.42
SMU_2115	Putative short-chain dehydrogenase	21	1.381	1	1	0.09
SMU_404	Hypothetical protein	26	1.378	1	1	0.23
SMU_209	Hypothetical protein	97	1.375	5	3	0.58
SMU_535	Putative glycerol phosphate synthase	34	1.368	2	1	0.1
SMU_1272	Putative histidyl-tRNA synthetase	29	1.367	2	1	0.07
SMU_1249	Hypothetical protein	208	1.365	6	2	0.21
SMU_1224	Putative dihydroorotate dehydrogenase	287	1.359	11	4	0.44
SMU_290	Conserved hypothetical protein	161	1.355	12	5	0.37
SMU_29	SAICAR synthetase	125	1.354	5	3	0.31
SMU_1443	Putative tributyrin esterase	26	1.354	2	1	0.19
SMU_37	IMP cyclohydrolase	344	1.353	18	5	0.32
SMU_735	Hypothetical protein	33	1.35	1	1	0.16
SMU_1925	Conserved hypothetical protein	82	1.349	4	2	0.27
SMU_284	Hypothetical protein	15	1.345	1	1	0.18
SMU_35	Glycinamide formyltransferase	40	1.337	2	1	0.14
SMU_537	Tryptophan synthase, beta subunit	157	1.334	12	3	0.2
SMU_1221	Orotate phosphoribosyltransferase	389	1.33	27	6	1.41
SMU_1091	Cell wall protein, WapE	714	1.325	30	8	0.54
SMU_1002	Putative DNA topoisomerase I	451	1.322	29	10	0.36
SMU.813	Putative transcriptional regulator	236	1.32	14	3	0.36
SMU_858	Aspartate transcarbamoylase	22	1.319	2	2	0.16
SMU_148	Alcohol-acetaldehyde dehydrogenase	2591	1.316	100	16	0.83
SMU_1223	Dihydroorotate dehydrogenase B	51	1.316	1	1	0.08
SMU_1421	Dihydrolipoamide acetyltransferase	40	1.314	4	3	0.2
SMU_458	ATP-dependent RNA helicase	78	1.309	3	2	0.1
SMU_690	Hypothetical protein	109	1.307	4	2	0.26
SMU_873	Homocysteine methyltransferase	64	1.306	4	4	0.13
SMU_472	Conserved hypothetical protein	234	1.303	10	4	0.34
SMU_910	Glucosyltransferase-S	463	1.298	31	14	0.29
SMU_100	Sorbose PTS system, IIB component	107	1.298	3	1	0.14
SMU_1977	Putative transcriptional regulator	59	1.298	3	2	0.64
SMU_1266	Glutamine amidotransferase HisH	47	1.298	3	1	0.13
SMU_1043	Phosphotransacetylase	670	1.296	30	5	0.66
SMU_2005	Putative adenylate kinase	383	1.291	15	4	0.72
SMU_1561	Potassium uptake system protein TrkB	201	1.291	8	2	0.24
SMU_1303	Putative dipeptidase	1176	1.285	46	9	0.79
SMU_1703	Conserved hypothetical protein	128	1.285	3	1	0.12
SMU_1437	UDP-N-acetylglucosamine 2-epimerase	306	1.284	14	5	0.36
SMU_881	Sucrose phosphorylase, GtfA	897	1.281	35	11	0.95
SMU_59	Phosphoribosylamine-glycine ligase	312	1.279	15	5	0.34

SMU_533	Anthranilate synthase, beta subunit	22	1.279	1	1	0.14
SMU_1869	Putative thioredoxin	513	1.277	24	4	1.86
SMU_1116	Hypothetical protein	525	1.276	22	9	1.01
SMU_577	Histidine kinase LytS	164	1.275	9	4	0.19
SMU_1214	Putative dihydroorotase	184	1.274	12	5	0.33
SMU_883	Dextran glucosidase DexB	148	1.272	12	2	0.14
SMU_335	Adenylosuccinate lyase	1406	1.271	56	14	1.54
SMU_1309	Glycerol dehydrogenase	1116	1.271	53	9	1.4
SMU_42	Conserved hypothetical protein	26	1.268	1	1	0.07
SMU_532	Putative anthranilate synthase	89	1.266	3	3	0.16
SMU_482	Putative RNA-binding Sun protein	54	1.266	2	2	0.1
SMU_1299	Acetate kinase	44	1.266	2	1	0.22
SMU_1434	Glycosyltransferase	158	1.265	9	3	0.2
SMU_1607	Exoribonuclease R (RNase R)	594	1.263	22	9	0.29
SMU_471	Conserved hypothetical protein	308	1.263	12	3	1.51
SMU_1732	Conserved hypothetical protein	156	1.263	8	3	0.37
SMU_1826	Putative aminotransferase	408	1.257	25	5	0.43
SMU_1765	Hypothetical protein	129	1.257	10	4	0.3
SMU_840	Hypothetical protein	19	1.256	2	1	0.37
SMU_2027	Putative transcriptional regulator	146	1.254	9	2	0.21
SMU_860	Carbamoylphosphate synthetase	1459	1.253	63	18	0.6
SMU_1065	Transcriptional regulator (GntR family)	153	1.253	8	3	0.34
SMU_1588	Putative hexosyltransferase	30	1.252	1	1	0.05
SMU_16	Putative amino acid permease	29	1.252	2	1	0.05
SMU_1269	Putative phosphoserine phosphatase	65	1.25	2	2	0.24
SMU_2127	Succinate aldehyde dehydrogenase	1381	1.246	58	10	1.06
SMU_328	Putative carbonic anhydrase	160	1.244	7	3	0.57
SMU_1205	Hypothetical protein	103	1.244	5	3	0.45
SMU_645	Putative oligopeptidase	22	1.242	2	2	0.08
SMU_1606	Putative SsrA-binding protein-like protein	88	1.241	4	2	0.3
SMU_915	Conserved hypothetical protein	121	1.24	5	3	0.5
SMU_49	Hypothetical protein	39	1.24	2	2	0.13
SMU_887	Galactose-1-P-uridyl transferase GalT	200	1.236	12	8	0.47
SMU_370	Putative ABC transporter	138	1.234	8	3	0.28
SMU_1058	Conserved hypothetical protein, SatD	20	1.233	1	1	0.1
SMU_412	Putative Hit-like protein	174	1.231	8	3	0.94
SMU_595	Putative dihydroorotate dehydrogenase	108	1.23	10	3	0.26
SMU.2073	Conserved hypothetical protein	32	1.23	1	1	0.42
SMU_1992	Putative tyrosyl-tRNA synthetase	519	1.229	19	5	0.46
SMU_1531	F0/F1 membrane-bound ATPase,	428	1.228	16	4	0.62
SMU_838	Glutathione reductase	312	1.225	18	3	0.25
SMU_608	Putative translation EF and RF	358	1.224	23	8	0.5
SMU_664	Putative ornithine acetyltransferase	16	1.222	1	1	0.06
SMU_58	Hypothetical protein	28	1.219	3	3	0.16
SMU_1651	Putative arsenate reductase	95	1.218	4	2	0.43
SMU_728	Putative oxidoreductase	47	1.216	3	2	0.18
SMU_1117	NADH oxidase	170	1.215	9	4	0.23
SMU_1859	Single-stranded DNA-binding protein	128	1.215	8	3	0.85
SMU_245	Negative regulator of competence	72	1.215	5	2	0.2
SMU_395	X-prolyl dipeptidyl peptidase	63	1.214	4	4	0.13
SMU_913	NADP- glutamate dehydrogenase	1967	1.212	83	11	1.41
SMU_787	Putative transcriptional regulator	652	1.212	25	7	0.47
SMU_1562	Putative potassium uptake protein TrkA	195	1.211	10	3	0.39
SMU_1396	Glucan-binding protein C, GbpC	180	1.211	8	4	0.18
SMU_1574	Conserved hypothetical protein	420	1.21	20	7	0.64
SMU_360	Extracellular G-3-P dehydrogenase	7226	1.204	363	13	4.31
SMU_1715	Conserved hypothetical protein	42	1.204	2	1	0.15

SMU_303	Conserved hypothetical protein	614	1.203	27	5	0.76
SMU_991	Putative ribonucleotide reductase	288	1.201	16	2	1.11
SMU_672	Isocitrate dehydrogenase	77	1.201	7	3	0.2
SMU_1071	Conserved hypothetical protein	75	1.201	5	2	0.17
SMU_137	Malolactic enzyme	1090	0.375	62	9	0.73
SMU_1984	Competence protein ComYC	42	0.462	2	2	0.49
SMU_140	Putative glutathione reductase	20	0.486	2	2	0.12
SMU_139	Conserved hypothetical protein	197	0.505	10	4	0.36
SMU_351	Conserved hypothetical protein	40	0.541	1	1	0.08
SMU_1699	Conserved hypothetical protein	32	0.542	3	3	0.25
SMU_587	Conserved hypothetical protein	16	0.543	1	1	0.08
SMU_1632	Putative MTA/SAH nucleosidase	104	0.545	2	1	0.11
SMU_1967	Single-stranded DNA-binding protein	47	0.558	1	1	0.19
SMU_1537	Glycogen biosynthesis protein GlgD	14	0.573	1	1	0.06
SMU_219	Putative transcriptional regulator	22	0.578	1	1	0.19
SMU.1345	Putative peptide synthetase	740	0.597	49	8	0.48
SMU.1410	Putative reductase	17	0.597	1	1	0.05
SMU_503	Hypothetical protein	23	0.599	2	1	0.12
SMU.1659	Conserved hypothetical protein	46	0.607	3	2	0.17
SMU_1405	Conserved hypothetical protein	1028	0.619	52	23	0.45
SMU_609	Putative 40K cell wall protein precursor	536	0.626	27	7	0.31
SMU_1340	Putative surfactin synthetase	873	0.631	39	15	0.25
SMU_1342	Putative bacitracin synthetase, BacA	2313	0.632	123	36	0.41
SMU.1343	Putative polyketide synthase	925	0.635	44	15	0.48
SMU_1344	Malonyl-CoA transacylase	416	0.635	20	6	0.39
SMU_1786	Undecaprenyl phosphate synthetase	211	0.636	10	3	0.32
SMU_1783	Putative prolyl-tRNA synthetase	863	0.639	37	8	0.42
SMU_1341	Putative gramicidin S synthetase	718	0.64	36	14	0.38
SMU_1347	Conserved hypothetical protein	711	0.646	41	9	0.46
SMU_1126	Putative pantothenate kinase	33	0.646	1	1	0.08
SMU_1415	N-acetyl-glucosamine phosphatases	53	0.657	2	2	0.2
SMU_2033	Hypothetical protein	24	0.669	1	1	0.04
SMU_1122	putative cytidine deaminase	22	0.679	1	1	0.19
SMU_826	Rhamnosyltransferase	68	0.68	2	1	0.07
SMU_956	Clp-like ATP-dependent protease	2452	0.692	92	21	1.64
SMU_1688	Extramembranal protein, DltD protein	121	0.692	6	2	0.17
SMU_301	Conserved hypothetical protein	38	0.692	1	1	0.12
SMU_400	Putative secreted esterase	18	0.697	1	1	0.07
SMU_1417	Oleoyl-acyl carrier protein thioesterase	592	0.706	29	4	0.72
SMU_1741	Putative malonyl-CoA transacylase	450	0.714	22	6	0.71
SMU_963	Conserved hypothetical protein	194	0.716	5	2	0.16
SMU_1803	Hypothetical protein	393	0.719	14	7	1.45
SMU_1904	Hypothetical protein	88	0.723	5	3	0.23
SMU_682	Hypothetical protein	64	0.723	5	3	0.08
SMU_546	Putative GTP-binding protein	1204	0.724	60	9	0.67
SMU_938	Putative phosphomevalonate kinase	70	0.725	4	3	0.24
SMU_1133	Transport system regulatory protein	250	0.726	10	6	0.84
SMU_1777	Ribonucleotide reductase protein, Nrd	93	0.728	3	1	0.17
SMU_751	Conserved hypothetical protein	371	0.729	18	13	0.58
SMU_1644	Hypothetical protein	597	0.733	30	5	0.85
SMU_08	Transcription-repair coupling factor	157	0.736	10	4	0.08
SMU_46	Hypothetical protein	38	0.736	1	1	0.11
SMU_1307	Conserved hypothetical protein	96	0.737	8	3	0.3
SMU_924	Thiol peroxidase	245	0.738	7	1	0.16
SMU_1428	Conserved hypothetical protein	137	0.738	10	3	0.29
SMU_453	Conserved hypoyhetical protein	68	0.741	3	2	0.15

SMU_469	Putative recombination protein U	31	0.744	1	1	0.12
SMU_317	Tetrahydrodipicolinate succinylase	686	0.746	28	5	0.94
SMU_401	Conserved hypothetical protein	95	0.746	2	2	0.35
SMU_1685	Conserved hypothetical protein	91	0.747	2	1	0.11
SMU_553	Conserved hypothetical protein	249	0.748	15	4	0.62
SMU_785	Putative shikimate kinase	218	0.749	13	4	1.07
SMU_1802	Conserved hypothetical protein	40	0.749	3	2	0.29
SMU_2098	Putative arginyl-tRNA synthase	501	0.75	28	13	0.71
SMU_385	Putative glycoprotein endopeptidase	195	0.75	7	4	0.52
SMU_1784	Putative Eep protein-like protein	119	0.75	5	3	0.19
SMU_1991	Penicillin-binding protein PBP1b	130	0.752	10	4	0.16
SMU_2071	Ribonucleotide reductase protein	89	0.752	3	2	0.25
SMU_1675	Cystathionine gamma-synthase	90	0.753	4	3	0.22
SMU_2162	Conserved hypothetical protein	21	0.753	1	1	0.14
SMU_251	Conserved hypothetical protein	385	0.754	25	6	0.42
SMU_1429	UDP-N-acetylmuramyl synthetase	142	0.754	9	2	0.11
SMU_159	Conserved hypothetical protein	69	0.754	3	1	0.17
SMU_901	Putative poly(A) polymerase	126	0.755	7	5	0.34
SMU_20	Cell shape-determining protein MreC	245	0.759	9	2	0.18
SMU_1427	Conserved hypothetical protein	166	0.759	5	2	0.15
SMU_1799	Adenylyltransferase	73	0.759	5	1	0.11
SMU_384	Hypothetical protein	113	0.76	7	3	0.51
SMU_777	P3-dehydroquinate dehydratase	31	0.76	1	1	0.11
SMU_972	UDP-N-acetylmuramate dehydrogenase	77	0.761	4	3	0.27
SMU_1168	Putative transcriptional regulator	28	0.761	1	1	0.13
SMU_743	Conserved hypothetical protein	105	0.762	7	2	0.18
SMU_1418	Putative coproporphyrinogen III oxidase	17	0.762	1	1	0.06
SMU_683	Putative ATP-binding protein	113	0.764	8	5	0.11
SMU_1577	Conserved hypothetical protein	168	0.766	12	9	0.17
SMU_623	Putative deacetylase	119	0.766	6	2	0.25
SMU.744	Cell division protein FtsY	328	0.767	11	3	0.15
SMU_470	Conserved hypothetical protein	19	0.767	1	1	0.13
SMU_1081	Conserved hypothetical protein	193	0.77	10	5	0.4
SMU_584	Putative arginine repressor	494	0.773	16	4	0.98
SMU_1484	Conserved hypothetical protein	139	0.774	9	6	0.36
SMU_2049	Conserved hypothetical protein	56	0.774	3	2	0.2
SMU_973	Spermidine/putrescine ABC transporter	34	0.774	3	2	0.13
SMU_1996	Isopentenyl monophosphate kinase	33	0.774	2	2	0.17
SMU_2050	Putative methyltransferase	16	0.774	1	1	0.08
SMU_984	Hypothetical protein	278	0.775	16	5	1.67
SMU_1663	Putative thymidylate kinase	257	0.775	12	4	0.55
SMU_1348	Putative ABC transporter	610	0.777	39	3	0.97
SMU_723	Putative calcium-transporting ATPase	337	0.777	26	9	0.28
SMU_253	D-alanyl-D-alanine carboxypeptidase	194	0.777	13	6	0.38
SMU_980	Putative PTS system	86	0.777	3	1	0.04
SMU_1382	Putative 3-isopropylmalate dehydratase	94	0.778	5	3	0.17
SMU_516	Conserved hypothetical protein	56	0.779	3	1	0.14
SMU_1689	D-alanine--poly(phosphoribitol) ligase	429	0.78	28	2	1.38
SMU_846	50S ribosomal protein L21	99	0.78	2	1	0.21
SMU_685	Hypothetical protein	69	0.78	5	2	0.38
SMU_1121	Hutative ABC transporter	485	0.781	20	4	0.29
SMU_1430	Putative cobyric acid synthase CobQ	84	0.781	5	2	0.2
SMU_755	Diacylglycerol transferase	20	0.781	1	1	0.1
SMU_759	Putative protease	293	0.783	12	3	0.25
SMU_43	Site-specific DNA-methyltransferase	68	0.783	4	3	0.15
SMU_946	Putative permease	48	0.783	2	1	0.09
SMU_556	Conserved hypothetical protein	22	0.783	1	1	0.09

SMU_187	Conserved hypothetical protein	164	0.785	10	4	0.35
SMU_165	Conserved hypothetical protein	78	0.786	3	2	0.19
SMU_1705	Hypothetical protein	116	0.787	5	2	0.27
SMU_161	Putative transcriptional regulator	47	0.787	2	1	0.09
SMU_06	Putative GTP-binding protein	335	0.788	15	5	0.37
SMU_07	Putative peptidyl-tRNA hydrolase	122	0.788	4	3	0.4
SMU_465	NAD(+) synthetase	484	0.789	18	4	0.81
SMU_1662	Putative DNA polymerase III, delta	66	0.789	3	2	0.17
SMU_1108	Conserved hypothetical protein	71	0.791	3	2	0.18
SMU_1491	PTS system lactose-specific EIICB	344	0.793	17	5	0.25
SMU_591	Hypothetical protein	28	0.793	3	1	0.06
SMU_1779	Putative RNA methyltransferase	69	0.794	6	1	0.05
SMU_829	Putative glycosyltransferase	67	0.794	8	4	0.21
SMU_1620	Phosphate starvation protein PhoH	51	0.794	5	1	0.08
SMU_2037	Trehalose-6-phosphate hydrolase TreA	862	0.795	39	5	0.41
SMU_717	Peptidoglycan synthesis protein MurM	169	0.796	14	3	0.24
SMU_1213	Putative 5'-nucleotidase precursor	36	0.796	1	1	0.04
SMU_870	Putative transcriptional regulator	267	0.799	11	3	0.29
SMU_1496	Galactose-6-phosphate isomerase	188	0.799	7	2	0.37
SMU_1179	Putative amino acid ABC transporter	98	0.799	8	2	0.26
SMU_1494	Tagatose-6-phosphate kinase	71	0.799	2	1	0.08
SMU.719	Conserved hypothetical protein	50	0.799	4	3	0.17
SMU_05	Conserved hypothetical protein	16	0.799	1	1	0.33

Table S2. Oligonucleotides used in this study

Primer Name	Sequence (5'- 3')	Purpose
SpxA2_BamH1_F	GGGGGATCCATGATTAAAATTTATACAATTTCAAGCTGTAC	Cloning of *spxA2* in pIBY35
SpxA2_Pst1_R	GGGCTGCAGTTATAAAGCTGCCCGTAAACGAGCTGCTG	Cloning of *spxA2* in pIBY35
BamH1_GFP_F	CGCGGATCCATGAGTAAAGGAGAAGA	Cloning of genes encoding for GFP and other GFP variants in pIB190
Pst1_GFP_R	GGGCTGCAGTTATTTGTATAGTTCATCCATGCC	Cloning of genes encoding for GFP in pIB190
Pst1_GFP-SpxA2_B (10 a.a)	GGGCTGCAGTTATAAAGCTGCCCGTAAACGAGCTGCTGTGTTTTTGTATAGTTCATCC	Cloning of genes encoding for GFP-SpxA2 (10 a.a) in pIB190
PstI-GFP-AVAA_R	GGGCTGCAGTTAAGCAGCAACAGCTTTGTATAGTTCATCCATGCC	Cloning of genes encoding for GFP-AVAA in pIBY35
PstI-GFP-RAAL_R	GGGCTGCAGTTATAAAGCTGCCCGTTTGTATAGTTCATCCATGCC	Cloning of genes encoding for GFP-RAAL in pIBY35
PstI-GFP-RAAD_R	GGGCTGCAGTTAATCAGCTGCCCGTTTGTATAGTTCATCCATGCC	Cloning of genes encoding for GFP-RAAD in pIBY35
PstI_GFP-RAVL_R	GGGCTGCAGTTATAAAACTGCCCGTTTGTATAGTTCATCCATGCC	Cloning of genes encoding for GFP-RAVL in pIBY35
PstI-GFP-AAL_R	GGGCTGCAGTTATAAAGCTGCTTTGTATAGTTCATCCATGCC	Cloning of genes encoding for GFP-AAL in pIBY35
PstI_GFP-EAAL_R	GGGCTGCAGTTATAAAGCTGCTTCGTATAGTTCATCCATGCC	Cloning of genes encoding for GFP-K/E-AAL in pIBY35
PstI_GFP-AAV_R	GGGCTGCAGTTAAACAGCTGCTTTGTATAGTTCATCCATGCC	Cloning of genes encoding for GFP-AAV in pIBY35
PstI_GFP-AAI_R	GGGCTGCAGTTAAATAGCTGCTTTGTATAGTTCATCCATGCC	Cloning of genes encoding for GFP-AAI in pIBY35
PstI_GFP-DEL _R	GGGCTGCAGTTATAATTCATCTTTGTATAGTTCATCCATGCC	Cloning of genes encoding for GFP-DEL in pIBY35
PstI_GFP-AIL_R	GGGCTGCAGTTATCCAGCTGCTTTGTATAGTTCATCCATGCC	Cloning of genes encoding for GFP-AIL in pIBY35
PstI_GFP-AVL_R	GGGCTGCAGTTATAATGCAACTTTGTATAGTTCATCCATGCC	Cloning of genes encoding for GFP-AVL in pIBY35
PstI_GFP-VAL_R	GGGCTGCAGTTATAAAACTGCTTTGTATAGTTCATCCATGCC	Cloning of genes encoding for GFP-VAL in pIBY35
PstI_GFP-ADL_R	GGGCTGCAGTTATAAATCTGCTTTGTATAGTTCATCCATGCC	Cloning of genes encoding for GFP-ADL in pIBY35
BamH1_GFP^{sf}_F	AATGGATCCATGTCAAAAGGAGAAGAGCT	Cloning of genes encoding for GFP^{sf} in pIBY35 and pASK-IBA43+
Pst1_GFP^{sf}_R	AATCTGCAGTTACTTATAAAGCTC	Cloning of genes encoding for GFP^{sf} in pIBY35 and pASK-IBA43+
Pst1_GFP^{sf}-AVAA_R	AATCTGCAGTTAAGCAGCAACAGCCTTATAAAGCTC	Cloning of genes encoding for GFP^{sf}-AVAA in pIBY35 and pASK-IBA43+
Pst1_GFP^{sf}-AAL_R	AATCTGCAGTTATAAAGCTGCCTTATAAAGCTC	Cloning of genes encoding for GFP^{sf}-AAL in pIBY35 and pASK-IBA43+
Pst1_GFP^{sf}-ETV_R	AATCTGCAGTTAAACAGTTTCCTTATAAAGCTC	Cloning of genes encoding for GFP^{sf}-ETV in pIBY35 and pASK-IBA43+
Pst1_GFP^{sf}-VTL_R	AATCTGCAGTTATAAAGTTACCTTATAAAGCTC	Cloning of genes encoding for GFP^{sf}-VTL in pIBY35 and pASK-IBA43+
Pst1_GFP^{sf}-VTK_R	AATCTGCAGTTATTTGGTCACCTTATAAAGCTC	Cloning of genes encoding for GFP^{sf}-VTK in pIBY35 and pASK-IBA43+
ClpX_ZBD del_invF	GTTCCAAAACCAAAAG	Cloning of genes encoding for ZBD deleted UA159 ClpX in pIB166
ClpX_ZBD del_invR	TGTGACATCATTGGTTC	Cloning of genes encoding for ZBD deleted UA159 ClpX in pIB166
pASK43_F	AGAGTTATTTTACCACTCCCT	Verification of clones in pASK-IBA43+
pASK43_R	CGCAGTAGCGGTAAACG	Verification of clones in pASK-IBA43+
pIB190_F	GGCTATTGGTGTTTATGGCTCTCTTGGTCGTC	Verification of clones in pIB190 and pIBY35
pIB190_R	CGCAGCGAGTCAGTGAGCGAGGAAG	Verification of clones in pIB190 and pIBY35
pIB166_F	GGAATAAGCGTTCGGTCAGT	Verification of clones in pIB166
pIB166_R	GGGATAAGAATGAGAGTATCAATGGC	Verification of clones in pIB166

Table S3. Strains and plasmids used in this study

Strain and plasmid	Description[a]	Reference and source
**Strains**		
S. mutans UA159	Wild type, serotype c	Ajdic et al., 2002
S. mutans IBSJ4	UA159 derivative Δ*clpX*	Tao et al., 2012
S. mutans IBS512	UA159 derivative Δ*clpX*	Bannerjee and Biswas, 2008
S. mutans IBSJ2	UA159 derivative Δ*clpC*	Tao et al., 2012)
S. mutans IBSJ5	UA159 derivative Δ*clpE*	Tao et al., 2012)
E. coli BL21	Wild type	This study
E. coli DH5α	Wild type	This study
**Plasmids**		
pIB190	A shuttle vector for protein expression in *S. mutans*, Ery^r	Biswas et al., 2008
pIBY35	A shuttle vector for protein expression in *S. mutans*, Ery^r	Gurung and Biswas, 2022
pIB166	A shuttle vector for protein expression in *S. mutans*, Chl^r	Biswas et al, 2014
pASK-IBA43+	Expression plasmid for protein expression and purification in *E. coli*	IBA-Lifesciences
pIBW28	pIBY35::*spxA2*, Ery^r	This study
pIBP6	pIB190::*gfp*, Ery^r	Jana et al., 2016
pIBW8	pIB190::*gfp-ntaarlraal*, Ery^r	This study
pIBW11	pIB190::*gfp-raal*, Ery^r	This study
pIBW40	pIB190::*gfp-eaal*, Ery^r	This study
pIBW21	pIB190::*gfp-raad*, Ery^r	This study
pIBW10	pIB190::*gfp-aal*, Ery^r	This study
pIBW44	pIB190::*gfp-aav*, Ery^r	This study
pIBW45	pIB190::*gfp-aai*, Ery^r	This study
pIBW54	pIB190::*gfp-ail*, Ery^r	This study
pIBW55	pIB190::*gfp-val*, Ery^r	This study
pIBW57	pIB190::*gfp-avl*, Ery^r	This study
pIBW77	pIB190::*gfp-adl*, Ery^r	This study
pIBW61	pIB190::*gfp-del*, Ery^r	This study
pIB1F10	pIBY35::sf*gfp*, Ery^r	This study
pIB1F18	pIBY35::sf*gfp-aal*, Ery^r	This study
pIB1F19	pIBY35::sf*gfp-etv*, Ery^r	This study
pIB1F23	pIBY35::sf*gfp-vtl*, Ery^r	This study
pIB1F22	pIBY35::sf*gfp-vtk*, Ery^r	This study
pIB1F13	pIB166::ClpX, Chl^r	Gurung and Biswas, 2022
pIB1F24	pIB166::$ClpX_{\Delta ZBD}$, Chl^r	Gurung and Biswas, 2022
pIB1F27	pASK43+::*sfgfp*, Amp^r	This study
pIB1F28	pASK43+::*sfgfp-avaa*, Amp^r	This study
pIB1F29	pASK43+::*sfgfp-aal*, Amp^r	This study
pIB1F31	pASK43+::*sfgfp-etv*, Amp^r	This study
pIB1F32	pASK43+::*sfgfp-vtl*, Amp^r	This study
pIB1F33	pASK43+::*sfgfp-vtk*, Amp^r	This study
pIB1F2	pET-Duet::*S. mutans clpX*, Amp^r	Gurung and Biswas, 2022
pIBP86	pET3a::*S. mutans clpP*, Amp^r	Jana et al., 2016

Kan^r , kanamycin resistance; Amp^r , ampicillin resistance; Ery^r , erythromycin resistance.
